# CMTM6 overexpression confers trastuzumab resistance in HER2-positive breast cancer

**DOI:** 10.1186/s12943-023-01716-y

**Published:** 2023-01-10

**Authors:** Fei Xing, Hongli Gao, Guanglei Chen, Lisha Sun, Jiayi Sun, Xinbo Qiao, Jinqi Xue, Caigang Liu

**Affiliations:** Department of Oncology, Innovative Cancer Drug Research and Engineering Center of Liaoning Province, Cancer Stem Cell and Translation Medicine Lab, Shengjing Hospital of China Medical University, Shenyang, 110022 China

**Keywords:** CMTM6, HER2+ breast cancer, Ubiquitination, Trastuzumab resistance

## Abstract

Human epidermal growth factor receptor 2-positive (HER2+) breast cancer is characterized by invasive growth, rapid metastasis and chemoresistance. Trastuzumab is an effective treatment for HER2+ breast cancer; however, trastuzumab resistance leads to cancer relapse and metastasis. CKLF-like MARVEL transmembrane domain-containing 6 (CMTM6) has been considered as a new immune checkpoint for tumor-induced immunosuppression. The role of CMTM6 in trastuzumab resistance remains unknown. Here, we uncover a role of CMTM6 in trastuzumab-resistant HER2+ breast cancer. CMTM6 expression was upregulated in trastuzumab-resistant HER2+ breast cancer cell. Patients with high CMTM6 expressing HER2+ breast cancer had worse overall and progression-free survival than those with low CMTM6 expression. In vitro, CMTM6 knockdown inhibited the proliferation and migration of HER2+ breast cancer cells, and promoted their apoptosis, while CMTM6 overexpression reversed these effects. CMTM6 and HER2 proteins were co-localized on the surface of breast cancer cells, and CMTM6 silencing reduced HER2 protein levels in breast cancer cells. Co-immunoprecipitation revealed that CMTM6 directly interacted with HER2 in HER2+ breast cancer cells, and CMTM6 overexpression inhibited HER2 ubiquitination. Collectively, these findings highlight that CMTM6 stabilizes HER2 protein, contributing to trastuzumab resistance and implicate CMTM6 as a potential prognostic marker and therapeutic target for overcoming trastuzumab resistance in HER2+ breast cancer.

**Supplementary Information:**

The online version contains supplementary material available at 10.1186/s12943-023-01716-y.

## Introduction

Globally, breast cancer (BC) has replaced lung cancer as the most commonly diagnosed cancer in women, with an estimated 2.26 million new cases in 2020 [1]. BC is the leading cause of cancer-related mortality in females [2]. Disease progression after therapy and metastatic disease are the underlying causes of death in the majority of patients.

BC originates in epithelial cells of the mammary glands. Based on gene expression profiling and molecular pathology, BC can be classified into four subtypes: luminal A, luminal B, human epidermal growth factor receptor 2 (HER2+), and basal-like tumors. HER2+ BC accounts for 15–20% of all BC cases [3]. HER2+ BC is biologically and clinically aggressive, resistant to chemotherapy and hormone therapy, and associated with disease relapse, metastasis, and poor prognosis [4]. There is a urgent need for understanding the mechanisms underlying HER2-driven aggressiveness and drug resistance in BC to inform the development of more efficacious treatment regimens.

HER2 is a transmembrane tyrosine kinase receptor that belongs to the human epidermal growth factor (EGF) receptor family [5]. HER2 can be activated in a ligand-dependent or independent manner. HER2 overexpression serves as an oncogenic driver in the progression of BC, promoting constitutive activation of downstream signaling cascades that induce cell proliferation through the Ras-mitogen-activated protein kinase (MAPK) pathway, or inhibit cell death through the phosphatidylinositol 3′-kinase (PI3K)/protein kinase B (Akt)/mammalian target of rapamycin (mTOR) pathway [6, 7].

Trastuzumab, a humanized HER2-specific antibody drug, has changed the treatment paradigm for patients with HER2+ BC. Trastuzumab was approved in 1998 as the first anti-HER2 target therapy in metastatic HER2+ invasive BC [8]. Trastuzumab is currently used for adults with node-negative or node-positive HER2+ BC alone or in combination with anthracycline- or taxane-based chemotherapy [9]. A recent meta-analysis of randomized trials in women with node-negative or node-positive operable HER2+ BC patients has shown that chemotherapy plus trastuzumab significantly reduced the absolute 10-year BC recurrence risks by 9.0% (95% CI 7.4 to 10.7; *p* < 0.0001) and 10-year BC mortality by 6.4% (4.9 to 7.8; *p* < 0.0001), compared with chemotherapy alone [10].

Trastuzumab acts by various mechanisms to inhibit cell growth, including prevention of HER2 dimerization, downregulation of the HER2 receptor by endocytic destruction of the receptor, accumulation of the cyclin-dependent kinase (CDK) inhibitor p27 to promote cell cycle arrest, induction of antibody-dependent cellular cytotoxicity, and inhibition of constitutive HER2 cleavage/shedding mediated by metalloproteases [11, 12]. Although trastuzumab provides clinical benefit to HER2+ BC patients, a substantial number of patients have primary or acquired resistance to trastuzumab, requiring alternative therapies [13]. The resistance rate of trastuzumab in HER2+ BC is 66–88% when used as a single agent and 20–50% when combined with chemotherapy [14, 15].

Many studies have investigated the potential mechanisms underlying trastuzumab resistance, and have shown that trastuzumab resistance is associated with downstream signal activation, tumor stem cell self-renewal, host immune regulation, and epigenetic effects [16–18]. Furthermore, the HER2 gene mutations can alter signal transmission through the PI3K/AKT cascade, weakening the inhibitory effect of trastuzumab on the PI3K/AKT pathway [18]. Moreover, expression of other tyrosine kinase receptors and proteins in cellular membranes may mask HER2 receptors and prevent specific binding of trastuzumab to HER2 through steric hindrance [19]. These mechanisms have mainly been recognized from in vitro studies, and their usefulness as predictive or prognostic factors in clinical studies is uncertain. Further investigation of the molecular mechanisms and potential molecular targets underlying trastuzumab resistance in HER2+ BC is critical for improving therapeutic effectiveness and patient prognosis.

The CKLF-like MARVEL transmembrane domain-containing member (CMTM) gene family was first reported in 2003, and encodes proteins that link classical chemokines and the transmembrane-4 superfamily [20]. CMTM6 is crucial for secretory-protein release and has multiple roles in the physiological and pathological processes. CMTM6 overexpression is associated with the molecular and clinical characteristics of malignancy and is linked to worse prognosis in glioma, oral squamous cell carcinoma, head and neck squamous cell carcinoma, lung adenocarcinoma, non-small cell lung cancer, and macrotrabecular massive hepatocellular carcinoma [21–27]. Conversely, CMTM6 has a suppressive role in colorectal cancer and ovarian cancer. Recent reports have identified CMTM6 as a new immune checkpoint in tumor-induced immunosuppression, suggesting that targeting CMTM6 may be a novel approach to overcome tumor immune escape. CMTM6 may reduce ubiquitination and increase the half-life of programmed death-ligand 1 (PD-L1) protein, preventing PD-L1 degradation, which impairs T cell function. CMTM6 knockdown (resulting from PD-L1 downregulation) significantly promotes tumor-specific T cell activity both in vivo and in vitro [28, 29].

The effects of CMTM6 on trastuzumab resistance are unknown. This study aimed to evaluate the role of CMTM6 in trastuzumab resistance in HER2+ BC and explored a strategy to overcome trastuzumab resistance by inhibiting CMTM6. Our findings identify CMTM6 as a potential prognostic factor and a novel therapeutic target against trastuzumab resistance in BC.

## Materials and methods

### Cell lines and cell culture

Human SKBR3 (HER2+), MCF-7 (ER+, PR+/−, HER2−), MDA-MB-231 (TNBC), and MDA-MB-468 (TNBC) BC cell lines and the trastuzumab-resistant JIMT-1 (HER2+) BC cell line were obtained from American Type Culture Collection (Manassas, VA, USA). The BT-474 (ER + HER2+) BC cell line was obtained from National Collection of Authenticated Cell Cultures (Shanghai, China). MCF 10A, the normal breast epithelial cell line, was obtained from Procell Life Science & Technology Co., Ltd. (Wuhan, China). SKBR3 cells were cultured in McCoy’s 5A medium (Thermo Fisher, Waltham, MA) containing 10% fetal bovine serum (FBS; Cellmax, China). MCF-7 and MDA-MB-468 cells were cultured in 10% FBS DMEM (Thermo Fisher). MDA-MB-231 cells were cultured in 10% FBS L-15 medium (Thermo Fisher). JIMT-1 cells were cultured in 20% FBS DMEM (Thermo Fisher). BT-474 cells were cultured in 10% FBS RPMI-1640 medium (Thermo Fisher). MCF 10A cells were cultured in specific epithelial culture medium (CL-0525, Procell Co., Ltd). All cells were cultured in a humidified environment with, or without (MDA-MB-231 cells), 5% CO_2_ at 37 °C until they were 50–80% confluency. Cells (1 × 10^6^) were cultured in 10 cm culture dishes for 24 h and exposed to trastuzumab (440 mg/ bottle) alone or combination with pertuzumab (420 mg/bottle, both from Roche, Basel, Switzerland). In addition, the cells were cultured in the presence or absence of an optimal dose of MG132 or cycloheximide (CHX, Sigma, St. Louis, USA).

### Bioinformatical analysis

Gene expression data of different types of cancers and the relevant non-tumor tissues were collected from the Cancer Genome Atlas (TCGA) (https://portal.gdc.cancer.gov/projects/) and the Genotype-Tissue Expression (GTEx) (https://www.gtexportal.org/home/index.html). After Log2 transformation, the relative levels of CMTM6 mRNA transcripts in each type of malignant tumor and non-tumor tissues were analyzed. Similarly, the gene expression matrix of 57 BC cell lines was extracted from the Cancer Cell Line Encyclopedia (CCLE) (https://portals.broadinstitute.org/ccle/about) database and the relative levels of CMTM6 mRNA transcripts in different BC cell lines were analyzed by ggplot2 (v3.3.3) in the R v4.0.3 software package. The potential relationship between CMTM6 and HER2 mRNA transcripts in BC tissues in TCGA (TCGA-BRCA) was analyzed by Spearman correlation analysis.

To understand the role of CMTM6 in HER2+ BC, the transcriptomic data of BC were extracted from TCGA-BRCA and stratified into the high or low CMTM6 group, based on the median value of CMTM6 mRNA transcripts. The gene sets involved in tumor-related malignant behaviors and HER2-related signaling pathways were selected and analyzed for ranking by the gene set enrichment analysis (GSEA) using JAVA software gsea-3.0.jar with a running parameter number of 1000 and a *p*-value of < 0.05.

### Tissue samples

Breast tissues were obtained from 76 patients with HER2+ BC and 6 patients with a non-BC disease at Shengjing Hospital of China Medical University and Cancer Hospital of China Medical University between June 2010 and June 2011. The BC specimens were collected by core needle biopsy (*n* = 20) or intraoperatively (*n* = 56). Patients who received a core needle biopsy were treated with preoperative neoadjuvant trastuzumab. Patients who underwent a surgery were treated with postoperative adjuvant trastuzumab. Trastuzumab resistance was defined as tumor progression when assessment at 2-3 months post neoadjuvant therapy, or as a new recurrence within 12 months of postoperative adjuvant therapy. Overall, 28 BCs were resistant to trastuzumab, and 48 BCs were sensitive to trastuzumab. In addition, 30 pairs of fresh BC and corresponding non-tumor breast tissue samples were collected for protein and RNA analyses.

### Immunohistochemistry (IHC)

Breast tumor and para-tumor tissues were fixed and paraffin-embedded. The sections (4 μm) were dewaxed and rehydrated, followed by antigen retrieval in pH 6.0 citrate buffer heating in a microwave for 10 min. The sections were incubated in 3% hydrogen peroxide solution and blocked with 5% bovine serum albumin (BSA). The sections were probed at 4 °C overnight with an appropriate concentration of primary antibodies against CMTM6 (HPA026980, Sigma-Aldrich), HER2 (10004-T60, Sino Biological), Ki67 (ab92742, Abcam), or Caspase-3 (19677-1-AP, Proteintech). After being washed, the bound antibodies were detected with horseradish peroxidase (HRP)-conjugated secondary antibody at room temperature for 30 min and visualized using 3,3′-Diaminobenzidine (DAB). The immune signals were observed under a light microscope (Olympus, Japan) in a blinded manner. Staining intensity was scored as: 3, strong (brown); 2, moderate (yellow brown); 1, weak (weak yellow); 0, negative (no staining). Staining extent was scored, according to the percentage of positive cells as: 1, ≤ 5%; 2, 6-25%; 3, 26–75%; and 4, ≥76% positive cells. The staining index was calculated by multiplying the staining intensity score by staining extent score, leading to a maximum staining index of 12. The cut off value was the staining index 3 and a specimen with a staining index of ≤3 was defined as low expression, whereas those with a staining index of > 3 were considered as high expression.

### Vector construction and transfection

The plasmid pcDNA3.1-HA-ubiquitin and the parental control vector were purchased from OBiO Technology (Shanghai). The plasmid pcDNA3.1-FLAG-HER2 was derived from pcDNA3.1-EF1a-mcs-3flag-CMV-EGFP and purchased form HANBIO (Shanghai).

The full length of CMTM6 cDNA sequence was amplified by PCR using the cDNA of MDA-MB-231 cells as the template, and the primers of *EcoRI*-CMTM6-F (5′-CCCgaattcgGATGGAGAACGGAGCGGTGTA-3′) and *KpnI*-CMTM6-R (5′-CGCggtaccTTAGGCATTAAGTGGCTCAGT-3′). After enzymatic digestions, the DNA fragment for CMTM6 expression was cloned into the *EcoRI* and *KpnI* sites of the plasmid of pCMV-Myc (a kindly gift from Professor Matsumiya Tomoh, Department of Bioscience and Laboratory Medicine, Hirosaki University Graduate School of Health Sciences). Finally, the plasmid sequence was confirmed by DNA sequencing. SKBR3 cells were transfected with the control plasmid or the plasmid for CMTM6 expression for 48 h for subsequent experiments.

JIMT-1, SKBR3 and HeLa cells were transfected with HA-ubiquitin, FLAG-HER2 and/or Myc-CMTM6 plasmids using Lipofectamine 3000 (Invitrogen), according to the manufacturer’s instructions.

To establish a stable CMTM6 silencing cell line, JIMT-1 cells were transduced with the lentivirus expressing control shRNA (NC) or CMTM6-specific shRNA (Sangon Biotech, Shanghai, China) at a multiplicity of infection of 10 and cultured in the presence of 5 μg/mL puromycin (Thermo Fisher) for 4 days. The efficiency of CMTM6 silencing was determined by Western blotting and quantitative reverse transcription-polymerase chain reaction (qRT-PCR). CMTM6-specific shRNA sequences were: shRNA#1, 5′-CCCAAGACAGTGAAAGTAATT-3′; shRNA#2, 5′-TGGAGAACGGAGCGGTGTACA-3′; shRNA#3, 5′-GCTGGCCTTCATCTGTGAAGA-3′.

### RNA extraction and qRT-PCR

Total RNA was extracted from cells and tissue samples using the Trizol reagent (Thermo Fisher) and reversely transcribed into complementary DNA with PrimeScript reverse transcriptase (Takara, Japan), according to the manufacturer’s instructions. The relative levels of targeted gene to control glyceraldehyde 3-phosphate dehydrogenase (GAPDH) mRNA transcripts were quantified by qRT-PCR using specific primers. The sequences of primers were CMTM6: F: 5′-TTTCCACACATGACAGGACTTC-3′, R: 5′-GGCTTCAGCCCTAGTGGTAT-3′; HER2: F: 5′-CAGGTGATGACTTCCAGCTCA-3′, R: 5′-CCCAGTGGCAGAAGGTCTTG-3′; and GAPDH: F: 5′-GAAGGTGAAGGTCGGAGTC-3′, R: 5′-GAGATGGTGATGGGATTTC-3′. The data were analyzed by 2^-ΔΔCt^.

### Western blot

Cells were lysed in radioimmunoprecipitation (RIPA) lysis buffer containing phenylmethane sulfonyl fluoride (PMSF) and protease and phosphatase inhibitors, and centrifuged. The protein concentrations in the cell lysate supernatants were measured using a Pierce BCA Protein Assay Kit (Thermo Fisher), according to the manufacturer’s instructions. Protein samples (30 μg) were separated by sodium dodecyl sulfate-polyacrylamide gel electrophoresis (SDS-PAGE) and transferred onto a polyvinylidene difluoride membrane (Millipore, Billerica, MA, USA). The membranes were blocked with 5% non-fat milk in Tris-buffered saline with 0.1% Tween-20 (TBST) for 2 h at room temperature and incubated overnight at 4 °C with an appropriate concentration of primary antibody against CMTM6 (HPA026980, Sigma-Aldrich), HER2 (10004-T60, Sino Biological), p-HER2 (R22909, Chengdu Zen Biotechnology), E-cadherin (20874-1-AP, Proteintech), N- cadherin (22018-AP-1, Proteintech), MEK (11049-1-AP, Proteintech), PI3K (20584-1-AP, Proteintech), AKT (51077-1-AP, Proteintech), ERK (11257-1-AP, Proteintech), β-actin (23660-1-AP, Proteintech), GAPDH (10494-1-AP, Proteintech), tubulin (2125, Cell Signaling Technology), according to the manufacturers’ instructions. After washing three times with TBST for 10 min each, the membranes were incubated with HRP-conjugated secondary antibodies (M21008, Abmart) for 1 h at room temperature, and visualized using an enhanced chemiluminescence system. The relative levels of protein expression were analyzed by densitometric scanning using ImageJ software.

### Cell counting kit-8 (CCK-8) assay

JIMT-1 or SKBR3 cells (5000 cells/well) were cultured in 96-well plates and treated in triplicate with trastuzumab at 0, 1, 2, 5, 10, 20, 50 and 100 μg/mL or 0, 0.5, 1, 2, 5, 10, 20, and 40 μg/mL for 72 h, respectively. During the last hour culture, the cells in each well were treated with 10 μL/well of CCK- 8 reagent (Dojindo, Japan) and their viability was determined by measuring the absorbance of individual wells at 450 nm using a microplate reader. The percentages of viable cells were calculated and dose-response curves were plotted.

### Wound healing and cell invasion assays

BC cells were cultured in 24-well plates up to 90% confluency. The monolayer cells were scraped using a sterile 200 μL pipette tip. After being washed, the cells were treated in triplicate with 10 μg/ml trastuzumab for 24 h and the wound areas were photoimaged at 0 h and 24 h, respectively. Cell invasion assays were performed in 24-well transwell plates (8 μm pore. Corning, USA). Briefly, BC cells were cultured in triplicate with 10 μg/ml trastuzumab in FBS-free medium in the top chamber that had been coated with Matrigel and the bottom chambers were added with the complete medium for 24 h. The cells onto the upper surface of the top chamber were removed with a cotton ball. The cells that had invaded into the lower surface of the top chamber were fixed with 0.5% glutaraldehyde and stained with 0.5% toluidine blue, followed by photoimaging under a light microscope (× 20). The invaded cells were enumerated in three fields of view/membrane.

### Co-immunoprecipitation assay

Cells were lysed in RIPA lysis buffer containing protease/phosphatase inhibitor. After centrifuged, the cell lysate samples were incubated at 4 °C for 4 h with anti-CMTM6 antibody (HPA026980, Sigma-Aldrich), anti-HER2 antibody (10004-T60, Sino Biological), or control immunoglobulin IgG (2 μg, sc-2025, Santa Cruz) and reacted with 20 μL Dynabeads (sc-2003, Santa Cruz Biotechnology, Dallas, TX) at 4 °C overnight with a gentle shake. After being washed with ice-cold PBS for three times to remove unbound antibodies, the bound immunocomplex on the beads was resolved by SDS-PAGE and analyzed by Western blot using anti-HER2 or anti-CMTM6, respectively.

### Immunofluorescence assay

BC cells (5 × 10^4^ cells/well) were cultured in 10% FBS medium on glass coverslips in 24-well plates for 24 h, washed with PBS for three times, fixed in 4% paraformaldehyde for 30 min. The cells were permeabilized using 0.5% Triton X-100 in PBS at room temperature for 1 min, and blocked with 5% BSA at 37 °C for 1 h. The cells were incubated overnight with anti-CMTM6 antibody (HPA026980, Sigma-Aldrich) and/or anti-HER2 antibody (60311-1-AP, Proteintech) at 4 °C. After being washed, the bound antibodies were reacted with a corresponding fluorophore-conjugated secondary antibody (ab150077, ab150115, Abcam) at room temperature for 1 h and nuclear-counterstained with 4′,6-diamidino-2-phenylindole (DAPI, D4054, UE, China). The immunofluorescent signals were obtained and photoimaged under a confocal laser-scanning microscope (Zeiss, LSM800).

### Terminal deoxynucleotidyl transferase dUTP nick end labeling (TUNEL) assay

The apoptotic cells were analyzed in situ by TUNEL assay using the TUNEL test kit (KeyGEN, Guangdong, China), according to the manufacturer’s instructions. Briefly, BC cells were cultured on glass coverslips in 12-well plates for 6 h and treated in triplicate with 10 μg/ml trastuzumab, followed by fixing in 4% paraformaldehyde at room temperature for 30 minutes. After being washed, the cells were permeabilized with 0.1% Triton X100 at room temperature for 5 min and reacted with TdT enzyme at 37 °C for 1 h. Subsequently, the cells were stained with Streptavidin TRITC for 30 minutes, and after being washed, the cells were nuclear-stained with DAPI. Finally, the fluorescent signals were captured and photoimaged under a fluorescence microscope.

### 5-ethynyl-2′-deoxyuridine (EdU) assays

The proliferation of BC cells was determined by EdU assay using the EdU test kit (KeyGEN, Guangdong, China), according to the manufacturer’s instructions. Briefly, BC cells were cultured in 12-well plates and treated in triplicate with 10 μg/ml trastuzumab for 24 h. The cells were fixed in 4% paraformaldehyde at room temperature for 30 minutes, washed three times with PBS, and permeabilized with penetrating solution at room temperature for 5 minutes. After being washed, the cells were stained with EdU working solution and nuclear-stained with DAPI. The EdU+ proliferative cells were observed under a fluorescence microscope.

### Xenograft tumor in nude mice

Animal experiments were approved by the Animal Experimentation Ethics Committee of Shengjing Hospital of China Medical University (Shenyang, China). Female nude mice at 4 weeks old, weighing 19–22 g were from SiPeiFu Biotechnology (Beijing, China) and housed in our specific pathogen-free facility. Individual mice were injected subcutaneously with 2 × 10^6^ cells into their mammary fat pads. When the tumor reached approximately 100 mm^3^, the mice were randomized (*n* = 4 per group) and treated intraperitoneally with the vehicle as the control group, trastuzumab (10 mg/kg body weight alone, as the Ttzm group) or the combination of same dose of trastuzumab and pertuzumab as the Ttzm+Ptzm group every 5 days for 4 times. The mice were monitored for their tumor growth and body weights every 5 days and the tumor volume was calculated by the formula of (L x W^2^)/2, where L and W are the longest and shortest diameters, respectively. The mice were euthanized after the last treatment, and their tumor tissues were dissected for immunohistochemistry and Western blot analyses.

The dissected xenograft tumor tissues were routine-fixed in 10% formalin (pH 7.0), and paraffin-embedded. The paraffin-embedded tissue sections (4 μm) were dewaxed, rehydrated and subjected to IHC using anti-Ki67, anti-caspase-3, anti-CMTM6 and anti-HER2, as described above.

### Statistical analysis

Statistical analyses were performed using GraphPad Prism 7 software (GraphPad Software). Data are presented as the mean ± standard deviation (SD) and were analyzed by the log-rank, χ^2^, Spearman’s rank correlation, two-sided Student’s t-, or two-tailed unpaired Student’s *t-*test (for two groups), or one-way analysis of variance (> 2 groups) where applicable. Correlations between variants were determined by the Pearson’s and Spearman’s r coefficients. A *P*-value of < 0.05 was considered statistically significant.

## Results

### CMTM6 is highly expressed in BC tissues and cell lines

**Fig. 1:**
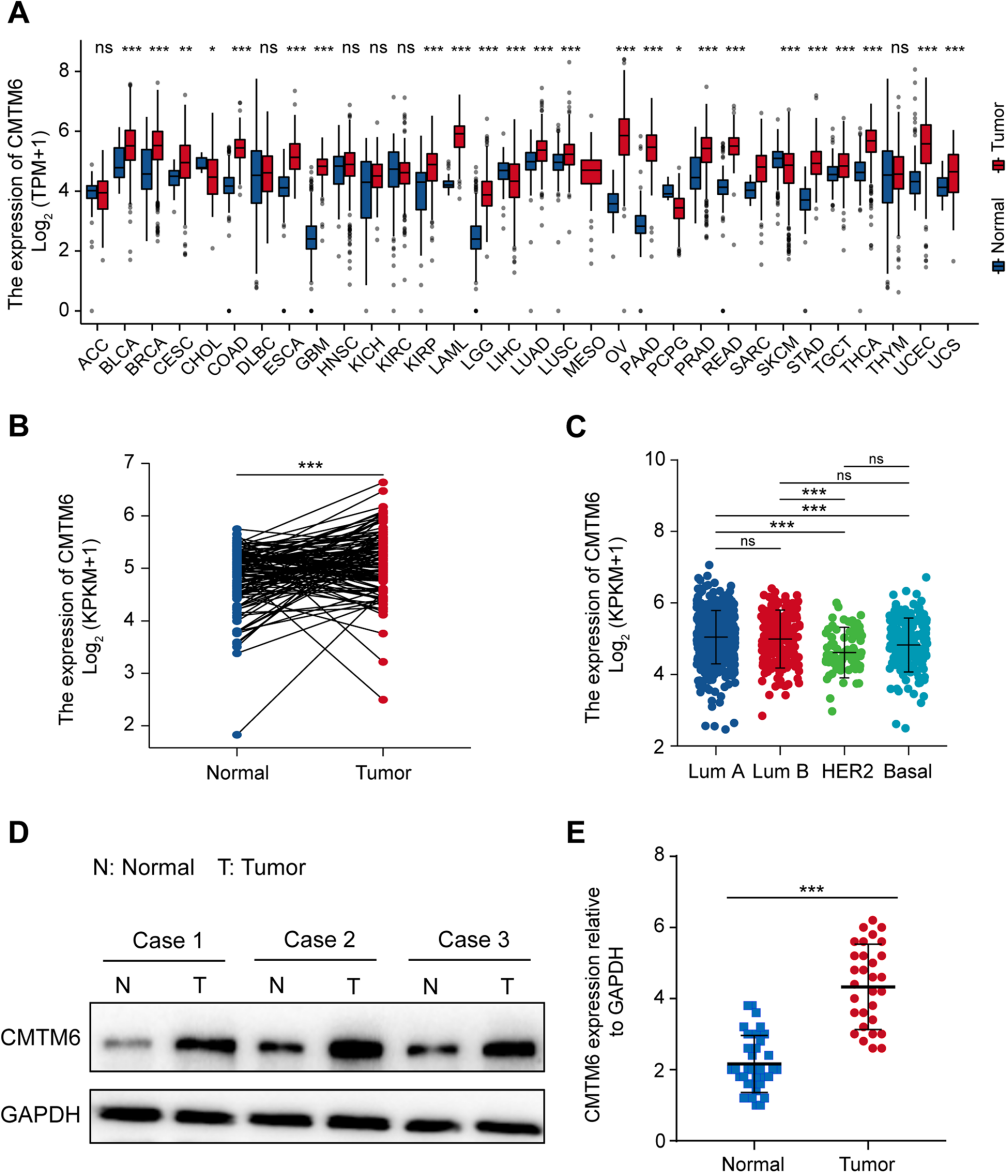
CMTM6 expression is upregulated in BC tissues and cell lines. **A** CMTM6 expression in different types of cancers (versus non-tumor tissues) in the TCGA and GTEx datasets. **B** CMTM6 expression in BC tissues and matched adjacent non-tumor tissues in the TCGA-BRCA dataset. **C** CMTM6 expression in different subtypes of breast cancers in the TCGA-BRCA datasets. **D** Western blot analysis of the relative levels of CMTM6 protein expression in fresh BC tissues and matched adjacent non-tumor tissues (*n* = 3). **E** qRT-PCR analysis of the relative levels of CMTM6 mRNA transcripts in fresh BC tissues and matched adjacent non-tumor tissues (*n* = 30). ***P < 0.001

To characterize the CMTM6 expression in human cancers, pan-cancer analysis of CMTM6 mRNA levels was performed in tumor tissues in the Genotype-Tissue Expression (GTEx) and the Cancer Genome Atlas (TCGA) datasets and corresponding non-tumor tissues from the TCGA dataset. CMTM6 mRNA transcripts were significantly higher in bladder urothelial carcinoma (BLCA), breast invasive carcinoma (BRCA), colon adenocarcinoma (COAG), esophageal carcinoma (ESCA), glioblastoma multiforme (GBM), kidney renal papillary cell carcinoma (KIRP), acute myeloid leukemia (LAML), brain lower-grade glioma (LGG), lung adenocarcinoma (LUAD), lung squamous cell carcinoma (LUSC), ovarian serous cystadenocarcinoma (OV), pancreatic adenocarcinoma (PAAD), prostate adenocarcinoma (PRAD), rectum adenocarcinoma (READ), stomach adenocarcinoma (STAD), thyroid carcinoma (THCA), uterine corpus endometrial carcinoma (UCEC), and uterine carcinosarcoma (UCS) than that in the corresponding non-tumor tissues (all *P* < 0.001, Fig. 1A).

To characterize the CMTM6 expression in BC tissues, CMTM6 mRNA transcripts in 112 BC tissues and 112 matched adjacent non-tumor tissues in the TCGA-BRCA dataset (Fig. 1B) were compared. CMTM6 mRNA transcripts in BC tissues were significantly higher than that in the corresponding non-tumor tissues (*P* < 0.001). Further analysis displayed that CMTM6 mRNA transcripts were detected in different subtypes of breast cancer tissues, and the relative levels of CMTM6 mRNA transcripts were slightly higher in luminal A and B subtypes than other subtypes (Fig. 1C). Western blotting and qRT-PCR analyses revealed that the relative levels of CMTM6 expression in fresh BC tissues were significantly higher than that in the matched adjacent non-tumor tissues (*P* < 0.001, Fig. 1D and E).

### Upregulated CMTM6 expression correlates with worse prognosis of patients with trastuzumab-resistant BC

**Fig. 2:**
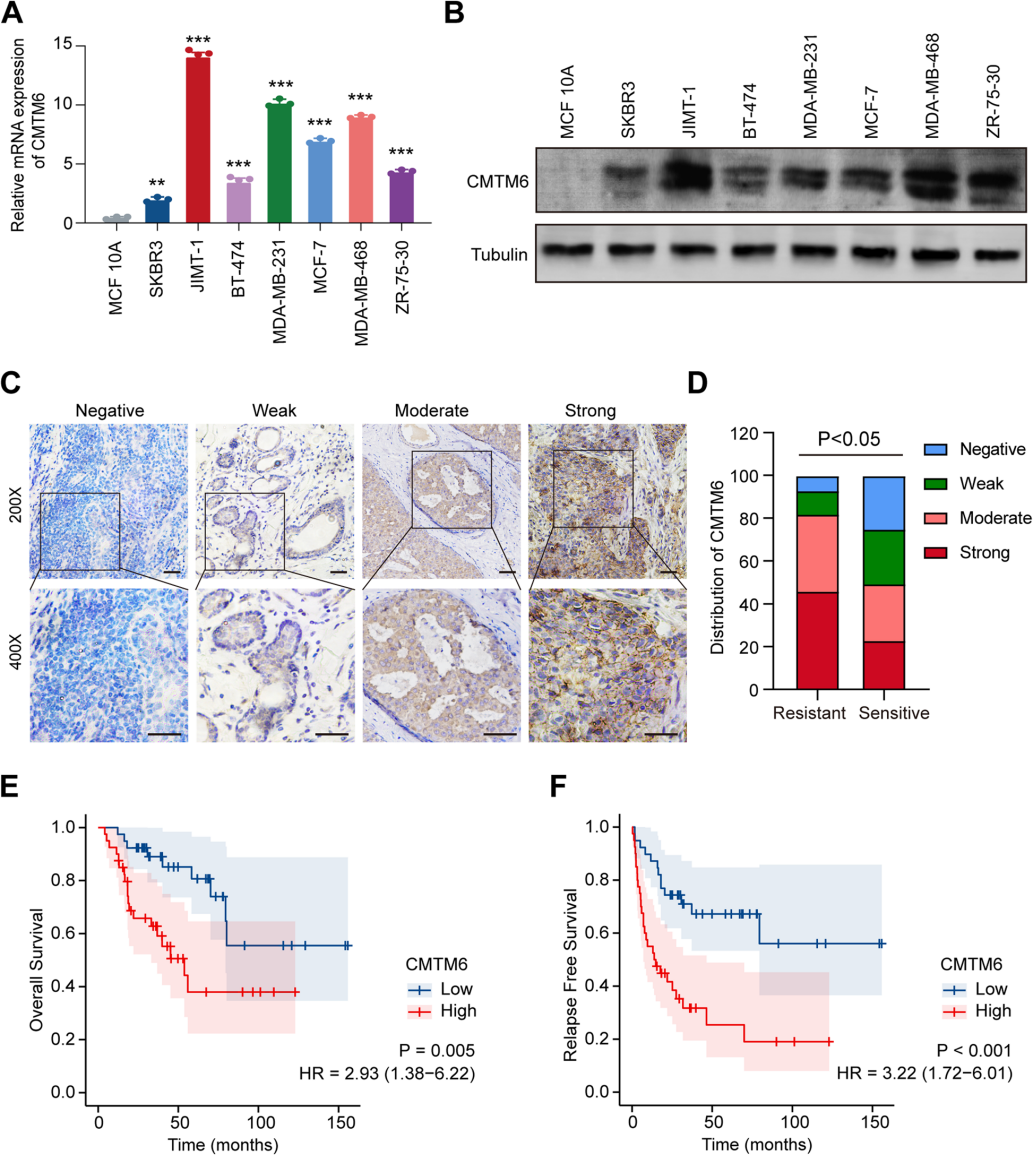
CMTM6 is upregulated in trastuzumab-resistant BC and correlates with poor prognosis. **A** qRT-PCR and **B** Western blot analyses of CMTM6 mRNA and protein expression in different BC cell lines. **C** Immunohistochemical analysis of CMTM6 protein expression in HER2+ BC tissues from trastuzumab-treated patients (scale bar, 50 μM). **D** Immunohistochemical analysis of CMTM6 protein expression in HER2+ BC tissues from trastuzumab-treated patients with or without relapse. **E, F** Kaplan–Meier analysis of overall survival (OS) and relapse-free survival (RFS) in trastuzumab-treated high or low CMTM6 expressing HER2+ BC patients

To investigate the clinical significance and biological role of CMTM6 in BC, the levels of CMTM6 mRNA transcripts in varying BC cell lines representing different subtypes of clinical BC in the Cancer Cell Line Encyclopedia (CCLE) dataset (https://portals.broadinstitute.org/ccle) were analyzed using the ggplot2 (v3.3.3) package in R (v4.0.3) (Fig. S1). Consistently, the levels of CMTM6 expression in SKBR3, JIMT-1, BT-474, MDA-MB-231, MCF-7, MDA-MB-468, and ZR-75-30 BC cells were higher than that in the non-tumor MCF 10A (Fig. 2A and B). Interestingly, very high levels of CMTM6 mRNA and protein expression were detected in the trastuzumab-resistant JIMT-1 (HER2+) BC cells while relatively lower levels of CMTM6 expression were observed in the trastuzumab-sensitive SKBR3 and BT474 BC cells, suggesting that up-regulated CMTM6 expression may be associated with trastuzumab resistance in HER2+ BC.

Next, 76 HER2+ BC tissues were collected from trastuzumab-treated patients and their CMTM6 protein expression was analyzed by IHC. CMTM6 protein expression was very low or nearly undetectable in 6 non-tumor breast tissues and negative, weak, moderate and strong CMTM6 expression were detected in 14, 15, 23 and 24 HER2+ BC tissues, respectively. (Fig. 2C-D). Stratification analyses uncovered that CMTM6 protein expression in HER2+ BC tissues from trastuzumab-treated patients was positively associated with clinical T stage (*P* = 0.007), clinical N stage (*P* = 0.039), metastasis (M classification, *P* = 0.023), pathological grade (*P* = 0.041), ER status (*P* = 0.016), relapse status (*P* < 0.001) and survival status (*P* < 0.001; Table 1). Further analyses indicated that trastuzumab-treated patients with high CMTM6 expressing HER2+ BC had a significant shorter overall survival (OS) and relapse free survival (RFS) than those with lower CMTM6 expressing HER2+ BC (Fig. 2E-F). Univariate (*P* = 0.015) and multivariate (*P* = 0.038) COX analyses unveiled that upregulated CMTM6 protein expression was one of the independent risk factors for the development of trastuzumab-resistance in patients with HER2+ BC (Tables 2 and 3). These data indicate that CMTM6 expression is upregulated in trastuzumab-resistant HER2+ BC and higher CMTM6 represents a potential predictor of poor prognosis in trastuzumab-treated patients with HER2+ BC.

**Table 1:** Association between CMTM6 expression and clinicopathological variables in HER2+ BC

Characteristic	Low expression of CMTM6	High expression of CMTM6	*P* value
n	29	47	
T stage, n (%)			0.007
T1	10 (13.2%)	9 (11.8%)	
T2	11 (14.5%)	25 (32.9%)	
T3	5 (6.6%)	5 (6.6%)	
T4	3 (3.9%)	8 (10.5%)	
N stage, n (%)			0.039
N0	11 (14.5%)	13 (17.1%)	
N1	14 (18.4%)	23 (30.3%)	
N2	3 (3.9%)	6 (7.9%)	
N3	1 (1.3%)	5 (6.5%)	
M stage, n (%)			0.023
M0	27(35.5%)	40 (52.7%)	
M1	2 (2.6%)	7 (9.2%)	
Pathologic stage, n (%)			0.041
Stage I	12 (15.8%)	2 (2.6%)	
Stage II	14 (18.4%)	31 (40.8%)	
Stage III	2 (2.6%)	11 (14.5%)	
Stage IV	1 (1.3%)	3 (3.9%)	
Age, n (%)			0.473
< =60	15 (19.7%)	22 (28.9%)	
> 60	14 (18.4%)	25 (32.9%)	
PR status, n (%)			0.222
Negative	12 (15.8%)	23 (30.3%)	
Positive	17 (22.4%)	24 (31.5%)	
ER status, n (%)			0.016
Negative	18 (23.7%)	10 (13.2%)	
Positive	11 (14.5%)	37 (48.7%)	
Relapse status, n (%)			< 0.001
No	26 (34.2%)	25 (32.9%)	
Yes	3 (3.9%)	22 (28.9%)	
Survival status, n (%)			< 0.001
Alive	20 (26.3%)	19 (25%)	
Dead	9 (11.8%)	28 (36.8%)

**Table 2:**
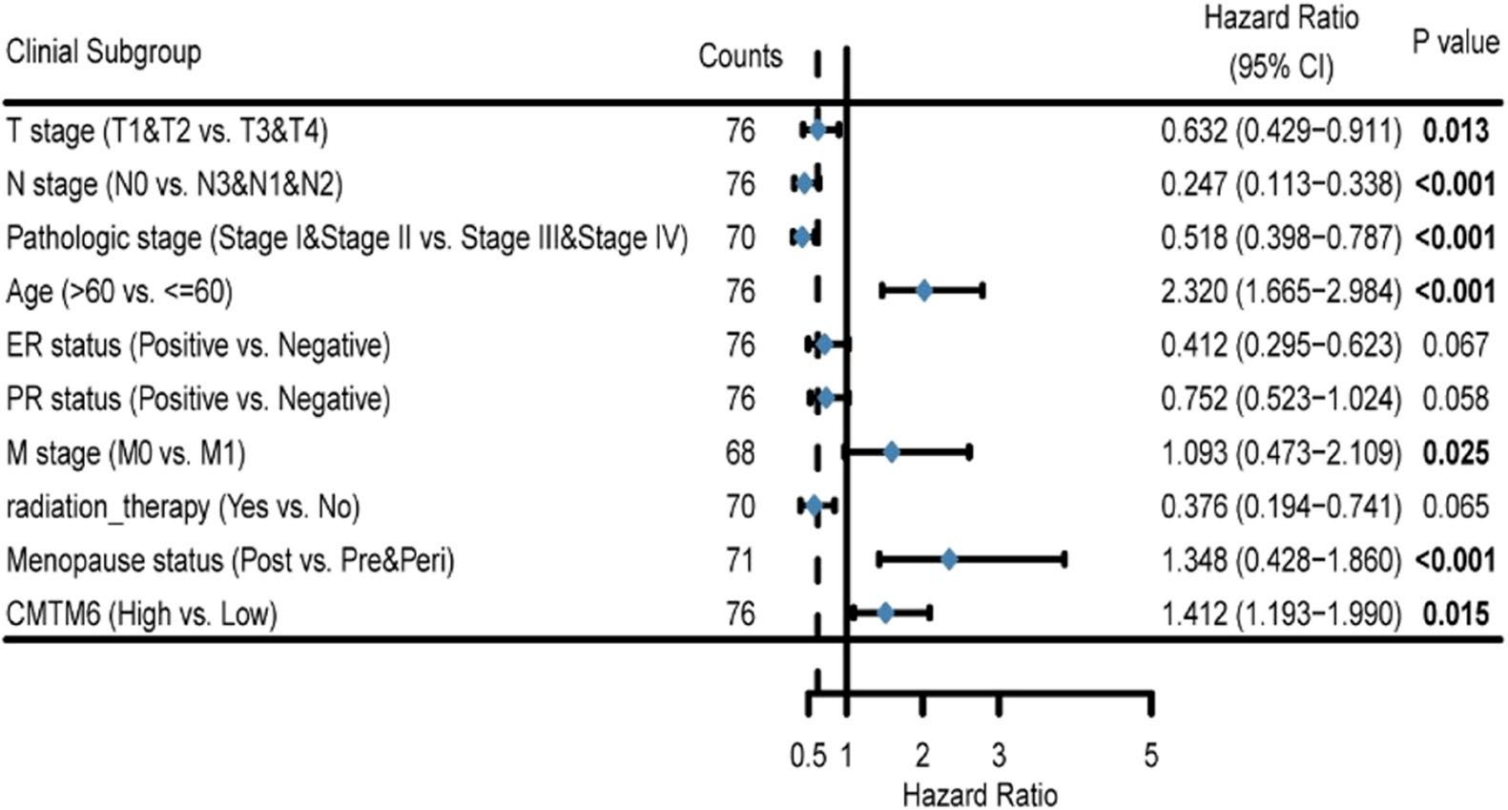
Univariate Cox regression analysis of different clinicopathological parameters and CMTM6 in HER2 + BC

**Table 3:**
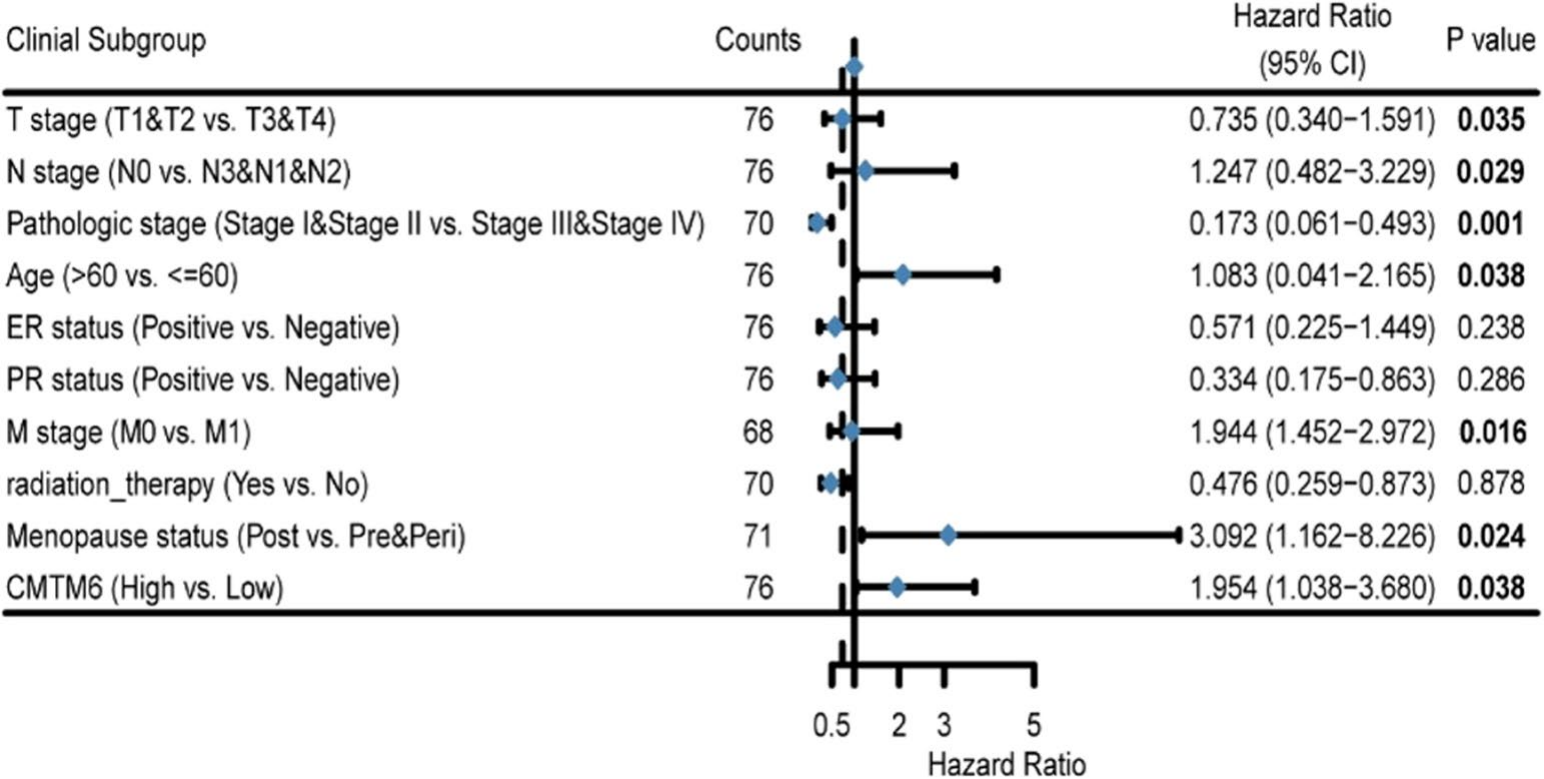
Multivariate Cox regression analysis of different clinicopathological parameters and CMTM6 in HER2 + BC

### CMTM6 promotes the survival, migration, invasion and trastuzumab resistance of BC cells in vitro

**Fig. 3:**
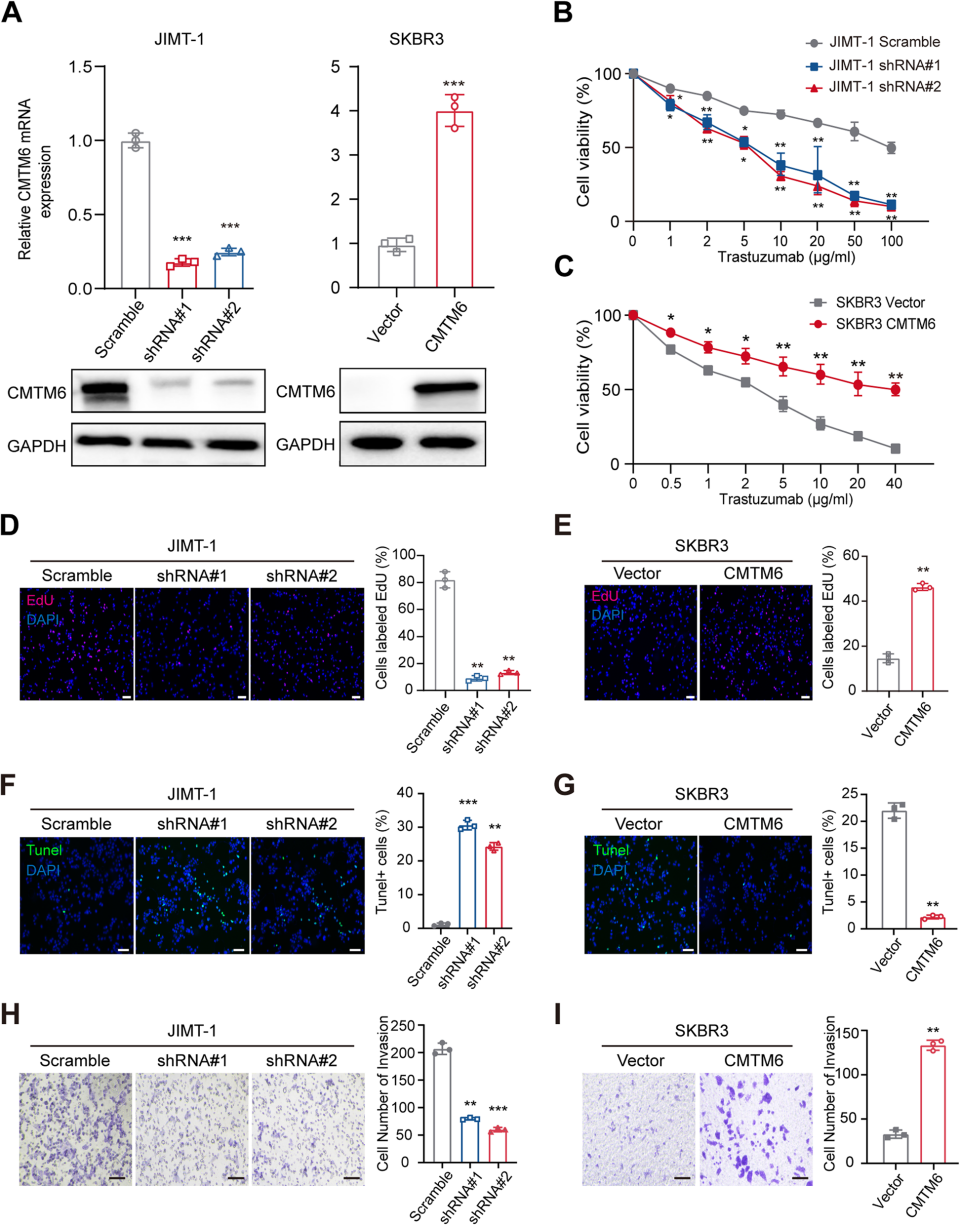
CMTM6 promotes the survival, migration, invasion and trastuzumab resistance of BC cells in vitro*.***A** qRT-PCR and Western blot validated CMTM6 silencing in JIMT-1 cells and CMTM6-overexpresstion in SKBR3 cells. Negative control (NC) JIMT-1 and SKBR3 cells were transduced with lentivirus for the control shRNA or transfected with the control plasmid, respectively. **B, C** CCK-8 assay determined the viability of the indicated BC cells following treatment with trastuzumab (0-100 μg/ml). **D, E** ethynyl-2′-deoxyuridine (EdU) analysis of the proliferation of CMTM6-silenced JIMT-1 cells, CMTM6 overexpressing SKBR3 cells, control JIMT-1 cells and SKBR3 cells following treatment with trastuzumab (10 μg/ml). (scale bar, 50 μM). **F, G** TUNEL analysis of apoptotic CMTM6-silenced JIMT-1 cells, CMTM6 overexpressing SKBR3 cells, control JIMT-1 and SKBR3 cells following treatment with trastuzumab (10 μg/ml). **H, I** Cell invasion assay revealed that CMTM6 silencing inhibited JIMT-1 cell invasion while CMTM6 overexpression enhanced SKBR3 cell invasion following treatment with trastuzumab (10 μg/ml). (scale bar, 50 μM). Data are representative images or expressed as the mean ± SD of each group from three independent experiments. *P < 0.05; **P < 0.01; ***P < 0.001

To investigate the regulatory role of CMTM6 in trastuzumab-resistant BC cells, trastuzumab-resistant (high CMTM6 expressing) JIMT-1 cells were transduced with lentivirus for the expression of control shRNA or CMTM6-specific shRNA to generate stable CMTM6 silencing cells while trastuzumab-sensitive (low CMTM6 expressing) SKBR3 cells were transfected with the control plasmid or CMTM6-expressing plasmid to induce CMTM6 overexpressing cells (Fig. 3A). The CCK-8 assays exhibited that CMTM6 silencing significantly increased the trastuzumab sensitivity of JIMT-1 cells, while CMTM6 overexpression significantly decreased trastuzumab sensitivity in SKBR3 cells, compared to the control NC (all *p* < 0.05, Fig. 3B-C). Consistently, a similar pattern of BC proliferation was detected by EdU assays in different BC cell lines (all *p* < 0.01, Fig. 3D-E). Hence, CMTM6 acted as an oncogenic factor to promote trastuzumab resistance and proliferation of BC cells.

To assess the effects of CMTM6 on BC survival, the effects of CMTM6 on apoptosis were evaluated in trastuzumab-treated (10 μg/ml) CMTM6-silencing JIMT-1, CMTM6 overexpressing SKBR3, control JIMT-1 and SKBR3 cells. The TUNEL assays displayed that CMTM6 silencing significantly increased the percentages of TUNEL+ apoptotic JIMT-1 cells while CMTM6 overexpression had opposite effects on the apoptosis of SKBR3 cells (both *p* < 0.01, Fig. 3F-G).

As CMTM6 expression is positively correlated with metastatic properties in BC [30, 31], we analyzed whether CMTM6 expression could be involved in the wound healing and invasion of BC cells. First, GSEA enrichment analysis indicated that signaling pathways related to cell migration, invasion and epithelial to mesenchymal transition (EMT) were positively enriched in high CMTM6 expressing BC in TCGA-BRCA dataset (Fig. S2A-B). Second, the effects of CMTM6 on BC cell migration and invasion were evaluated in trastuzumab-treated (10 μg/ml) CMTM6-silenced JIMT-1, CMTM6 overexpressing SKBR3, and control JIMT-1 and SKBR3 cells. Cell invasion assays exhibited that CMTM6 silencing decreased the number of invaded JIMT-1 cells, relative to that of the control JIMT-1 cells while CMTM6 overexpression increased the number of invaded SKBR3 cells, compared to that of the control SKBR3 cells (*p* < 0.01, Fig. 3H-I). A similar pattern of wound healing was observed in different BC cell lines (both *p* < 0.01, Fig. S2C-D). Collectively, these data imply that CMTM6 enhances the malignant behaviors of BC cells in vitro.

### CMTM6 interacts with the HER2 signaling pathway in BC tissues and cells

**Fig. 4:**
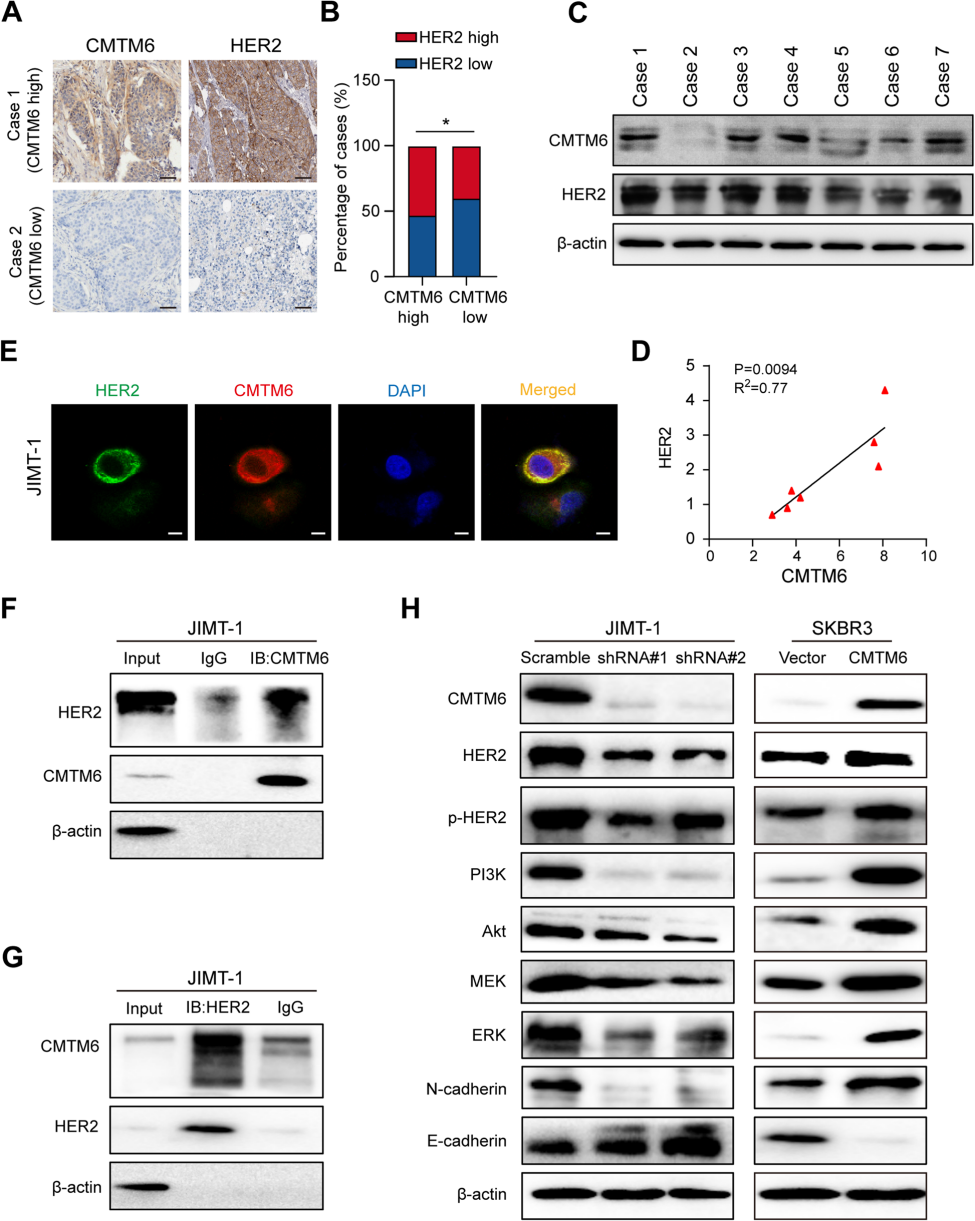
CMTM6 directly interacts with HER2 and enhancing the HER2 signaling in BC cells. **A** IHC analysis of HER2 protein expression in BC tissues with low or high CMTM6 protein expression (scale bar, 50 μM). **B** Association between CMTM6 and HER2 protein expression in BC tissues. **C** Western blot analysis of CMTM6 and HER2 protein levels in BC tissues. **D** Correlation between CMTM6 and HER2 protein levels in BC tissues. Data are mean ± SEM. **E** Confocal microscopy analysis of the subcellular co-localization of CMTM6 (green) and HER2 (red) in JIMT-1 cells, with DAPI nuclear staining (blue) (scale bar, 10 μM). **F, G** Co-immunoprecipitation revealed the direct interaction between endogenous CMTM6 and HER2 proteins in JIMT-1 cells. **H** Western blot analysis of CMTM6, HER2, p-HER2, PI3K, AKT, MEK, ERK, N-cadherin and E-cadherin protein levels in CMTM6-silenced JIMT-1, CMTM6 overexpressing SKBR3, control JIMT-1 and SKBR3 cells. Data are representative images of each group of cells from three separate experiments

Previous studies suggest that trastuzumab suppresses the oncogenic functions of HER2 receptors in HER2+ tumors and attracts immune cells to the HER2+ tumor environment [32, 33]. This implies that CMTM6 may modulate activation of the HER2 signaling in BC. To investigate the molecular mechanisms underlying the action of CMTM6 in trastuzumab resistance in BC tissues, the relationship between ERBB2 and CMTM6 expression in TCGA-BRCA datasets was analyzed by GSEA enrichment analysis. CMTM6 expression was positively correlated with ERBB2 expression, the ERBB2, the EGF-EGFR, the MAPK, the PI3K-AKT and the PI3K-AKT-mTOR signaling pathways (Fig. S3A-B). IHC and Western blot analyses revealed that HER2 expression was positively correlated with CMTM6 expression in 7 HER2+ BC tissues (*r* = 0.910, *P* = 0.004; Fig. 4A-D). Immunofluorescence and confocal microscopy unveiled that HER2 and CMTM6 protein expression were co-localized on the cell surface membrane of JIMT-1 cells (Fig. 4E). Co-immunoprecipitation exhibited that anti-CMTM6 precipitated HER2 protein and anti-HER2 also precipitated CMTM6 protein, an evidence of direct interaction between CMTM6 and HER2 proteins in JIMT-1 cells (Fig. 4F-G). Western blot displayed lower levels of HER2 protein expression and phosphorylation and the levels of PI3K, AKT, MEK, ERK, and N-cadherin, but slightly higher levels of E-cadherin expression in the CMTM6-silenced JIMT-1 cells, compared to NC, while higher levels of HER2 protein expression and phosphorylation and the levels of PI3K, AKT, MEK, ERK, N-cadherin expression, but slightly lower levels of E-cadherin expression were detected in the CMTM6 overexpressing SKBR3 cells, compared to the NC (Fig. 4H). Together, these data indicated that CMTM6 directly interacted with HER2 protein to enhance the HER2-related signaling and malignant behaviors of BC cells.

### CMTM6 stabilizes HER2 protein by inhibiting HER2 ubiquitination in BC cells

**Fig. 5:**
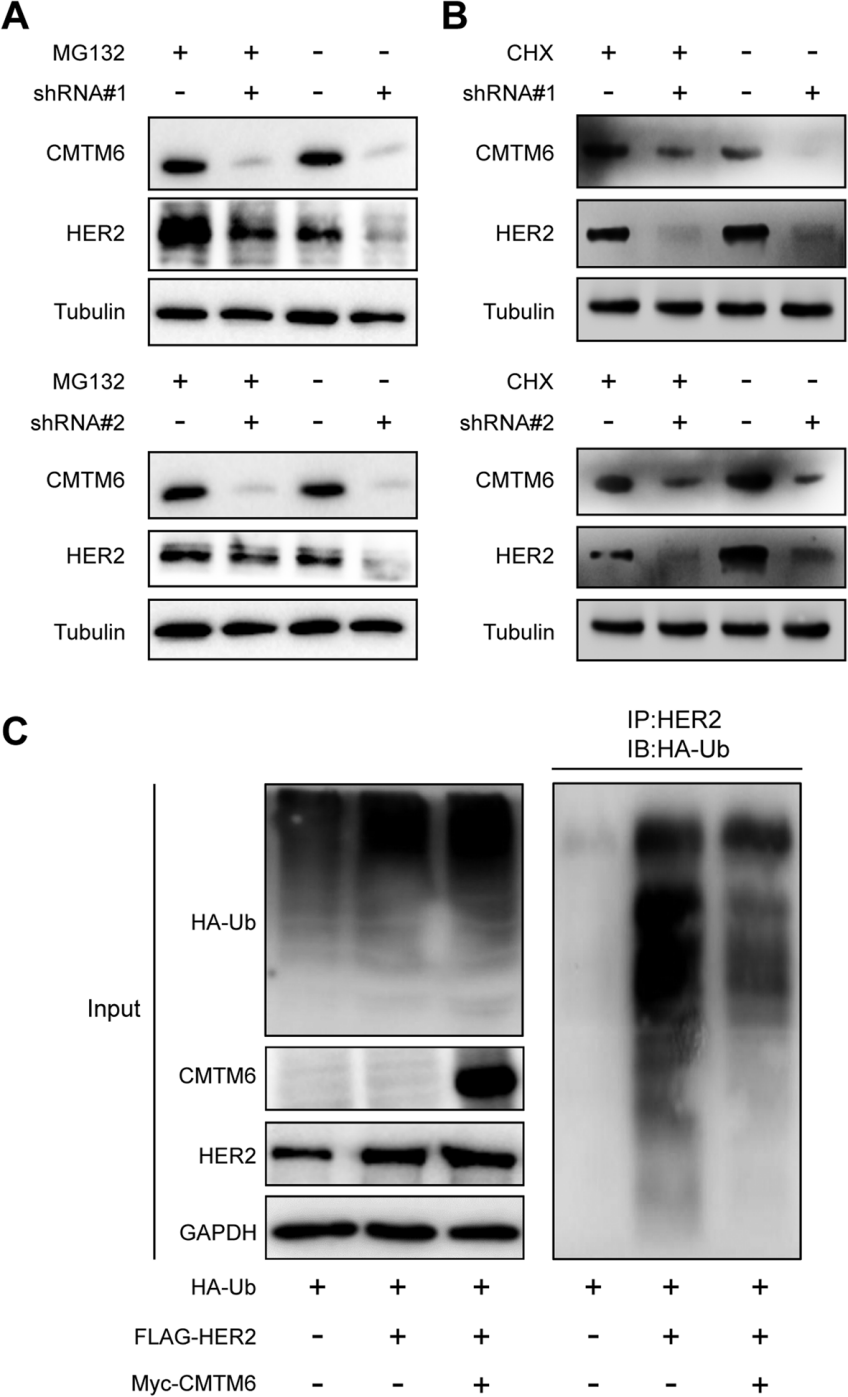
CMTM6 stabilizes the HER2 protein by inhibiting its poly-ubiquitination in BC cells. **A** Western blot analysis of CMTM6 and HER2 protein levels in CMTM6-silenced JIMT-1 cells after treatment with 10 μM MG132 or vehicle control for 24 h. **B** Western blot analysis of CMTM6 and HER2 protein levels in CMTM6-silenced JIMT-1 cells after treatment with 10 μM CHX or vehicle control for 24 h. **C** CMTM6 overexpression decreased HER2 poly-ubiquitination. HeLa cells were co-transfected with the FLAG-HER2 plasmid or combined with Myc-CMTM6 plasmid, together with the HA-Ub plasmid for 24 h and treated with 10 μM MG132 for another 24 h. The cell lysates were immunoprecipitated with anti-HER2 and immunoblotted with anti-HA antibody to determine HER2 poly-ubiquitination

CMTM6 can modulate the stability of PD-L1 by reducing its ubiquitination [24, 29], and HER2 is degraded by proteolytic ubiquitination [15, 34]; therefore, CMTM6 may enhance the stability of HER2 protein in BC cells. Western blot displayed that compared with the control NC cells, CMTM6 silencing decreased HER2 protein levels in JIMT-1 cells, which was reversed by treatment with a proteasome inhibitor of MG132 (Fig. 5A), but not with cycloheximide (CHX, a protein synthesis inhibitor, Fig. 5B). These data imply that CMTM6 affects HER2 protein levels through a post-translational mechanism. A ubiquitin-based immunoprecipitation assay unveiled a remarkable decrease in the levels of HER2 poly-ubiquitination in CMTM6 overexpressing HeLa cells, relative to the control NC (Fig. 5C). Thus, CMTM6 inhibited HER2 ubiquitination to stabilize HER2 protein in BC cells.

### CMTM6 promotes the growth of trastuzumab-resistant BC by increasing HER2 protein in BC tumors *in mice*

**Fig. 6:**
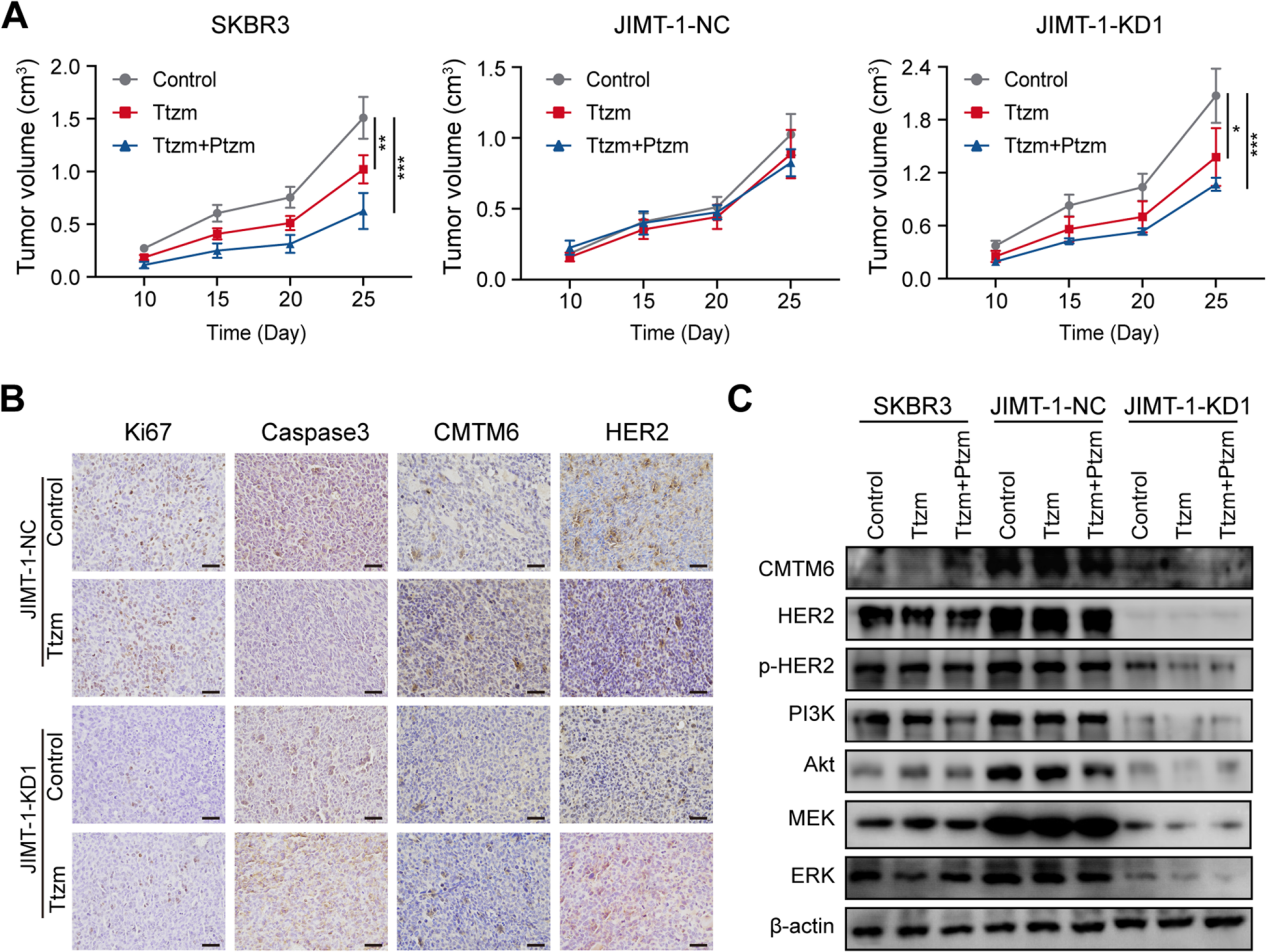
CMTM6 promotes the growth of trastuzumab-resistant BC by preserving HER2 protein and relative signaling in BC tissues in vivo. Female node mice were injected with SKBR3, CMTM6-silenced JIMT-1 or JIMT-1 cells to establish xenograft tumors and when the tumors reached at 100 mm^3^, the tumor-bearing mice were randomized and treated with vehicle (Control), trastuzumab (Ttzm, 10 mg/kg body weight every 5 days for 4 times) or combination of trastuzumab and Pertuzumab (Ttzm+Ptzm, *n* = 4 per group). **A** The dynamic growth of tumors. **B** IHC analysis of CMTM6, HER2, Ki67, and Caspase-3 protein expression (scale bar, 50 μM). **C** Western blot analysis of the relative levels of CMTM6, HER2, p-HER2, PI3K, AKT, MEK, ERK protein levels in the indicated tumors. Data are presentative images of each group from at least three separate experiments. *P < 0.05; **P < 0.01; ***P < 0.001

To further explore the potential effects of CMTM6 on the growth of trastuzumab-resistant BC, female node mice were injected with SKBR3, CMTM6-silenced JIMT-1 or control JIMT-1 cells into their mammary gland fat pads to induce xenograft tumors. When the induced tumors reached at 100 mm^3^, the tumor-bearing mice were randomized and injected intraperitoneally with the vehicle as the control, trastuzumab alone (Ttzm group) or the combination of trastuzumab and pertuzumab (Ttzm+Ptzm group) and their tumor growth was monitored longitudinally **(**Fig. 6A). Evidently, compared with the control group, treatment with trastuzumab alone significantly inhibited the growth of SKBR3 and CMTM6-silencing JIMT-1 tumors, but not the high CMTM6 expressing control JIMT-1 tumors, and treatment with pertuzumab enhanced the therapeutic effect of trastuzumab on the growth of SKBR3 and CMTM6-silencing JIMT-1 tumors (*p* < 0.001, *P* < 0.05). IHC exhibited that compared with the control JIMT-1 tumors, CMTM6, HER2 and Ki67 protein expression obviously decreased, and caspase-3 protein expression increased in the CMTM6-silenced JIMT-1 tumors (Fig. 6B). Finally, Western blot revealed that compared with the JIMT-1 tumors, the relative levels of CMTM6, HER2, p-HER2, PI3K, AKT, MEK and ERK protein expression were reduced in the CMTM6-silenced JIMT-1 tumors, particularly after trastuzumab treatment (Fig. 6C). These data further support the notion that CMTM6 promotes trastuzumab resistance in BC by stabilizing HER2 protein and enhancing its downstream signaling.

## Discussion

This study evaluated the role of CMTM6 in trastuzumab resistance and explored the potential mechanisms underlying the action of CMTM6 in trastuzumab resistance in HER2+ BC. Here, we show that patients with high CMTM6 expressing HER2+ BC have a worse prognosis than those with low CMTM6 expression following treatment with trastuzumab. In vitro experiments revealed that CMTM6 mRNA and protein levels were the highest in the trastuzumab-resistant JIMT-1 (HER2+) BC cells and lower in the trastuzumab-sensitive SKBR3 and BT-474 BC cells. CMTM6 silencing significantly increased trastuzumab sensitivity of JIMT-1 cells, while CMTM6 overexpression decreased trastuzumab sensitivity in SKBR3 cells. Furthermore, CMTM6 enhanced the malignant behaviors of BC cells by supporting their survival, proliferation, wound healing and invasion in vitro. Mechanistically, CMTM6 directly interacted with HER2 and limited HER2 ubiquitination to stabilize HER2 protein in BC cells. More importantly, treatment with trastuzumab alone or combination with Pertuzumab inhibited the growth of CMTM6-silancing JIMT-1 tumors, but not high CMTM6 expressing JIMT-1 tumors in mice. These findings further evidenced that CMTM6 enhanced trastuzumab resistance to promote the growth of HER2+ BC by enhancing the HER2-related signaling. Therefore, high CMTM6 expression may be a predicting factor for the prognosis of HER2+ BC and patients with high CMTM6 expressing HER2+ BC may require more aggressive treatments, other than trastuzumab.

Targeted anti-HER2 therapies are the key strategies for early and advanced HER2+ BC. Trastuzumab is the cornerstone drug for anti-HER2 treatment because it is safe, tolerable and efficacious. However, many patients develop trastuzumab resistance within 1 year following trastuzumab treatment, requiring the use of additional therapies [35]. It is essential to explore the mechanisms underlying trastuzumab resistance and identify biomarkers to predict the sensitivity to trastuzumab for individualized treatment in clinic. Other currently approved anti-HER2 drugs for HER2+ BC include pertuzumab, trastuzumab emtansine (T-DM1), and lapatinib. Pertuzumab is a humanized HER2 monoclonal antibody that recognizes the extracellular region of HER2 to inhibit HER2/HER3 heterodimerization, and T-DM1 combines trastuzumab and anti-microtubule agent DM1 [36]. After binding to the HER2 receptor on the surface of tumor cells, the receptor-T-DM1 complex is internalized into cells, and anti-microtubule drugs exert anti-tumor effects [37]. Lapatinib, neratinib, and pyrotinib are small HER2 tyrosine-kinase inhibitors that can bind to the ATP site of the receptor intracellular domain, block HER2 dimerization, interfere with signal transduction related to tumor cell proliferation and growth, and display excellent efficacy for HER2+ BC [38].

Here, we demonstrate, for the first time, that CMTM6 participates in trastuzumab resistance by directly interacting with HER2 protein and maintaining its cell surface expression. We speculate that this effect may be achieved by inhibiting HER2 ubiquitination, similar to the function of CMTM6 prolonging the half-life of PD-L1 [4]. We postulate that the binding site of CMTM6 may be located in the extracellular region of the HER2 protein, indicating that tyrosine-kinase inhibitors of HER2, such as rapamycin, lapatinib, and pyrotinib, which target the intracellular region of HER2, may be effective against trastuzumab resistance.

Prior evidence suggests that the expression of PD-L1 and HER2+ is closely correlated in HER2+ cancers. Trastuzumab significantly upregulates PD-L1 expression in HER2+ cancer cells by activating the NF-ĸB signaling to promote pro-inflammatory cytokine production. Induction of PD-L1 silencing can enhance trastuzumab efficacy in HER2+ cancers. Combination of trastuzumab and targeted immune checkpoint drugs can effectively overcome trastuzumab resistance in HER2+ tumors. Dual inhibition of HER2 and PD-L1 successfully enhances the anti-tumor effect of anti-HER2 monotherapy in HER2+ tumor cells. Inhibition of HER2 expression down-regulates PD-L1 expression in HER2-overexpressing BC cells. CMTM6 is a critical regulator of PD-L1 stability in a broad range of cancer cells, and CMTM6 stabilizes PD-L1 protein to promote tumor immune escape [28]. Currently, there is no information on the impact of CMTM6 on HER2-targeted therapy. Here, for the first time, we report that CMTM6 protects HER2 protein from its degradation, contributing to the maintenance of trastuzumab resistance in HER2+ BC. Considering the relationship among HER2, PD-L1, and CMTM6, we speculate that CMTM6 may act as a upstream regulator of HER2 and PD-L1. These data suggest that CMTM6 inhibition may alleviate trastuzumab resistance and promote the efficacy of anti-HER2 therapy, highlighting the need to develop small-molecule inhibitors of CMTM6 as potential therapeutic strategies for the treatment of trastuzumab-resistant HER2+ BC. We are interested in further investigating the molecular mechanisms by which CMTM6 regulates trastuzumab resistance and potential relationship among HER2, PD-L1 and CMTM6 in BC.

## Conclusion

**Fig. 7:**
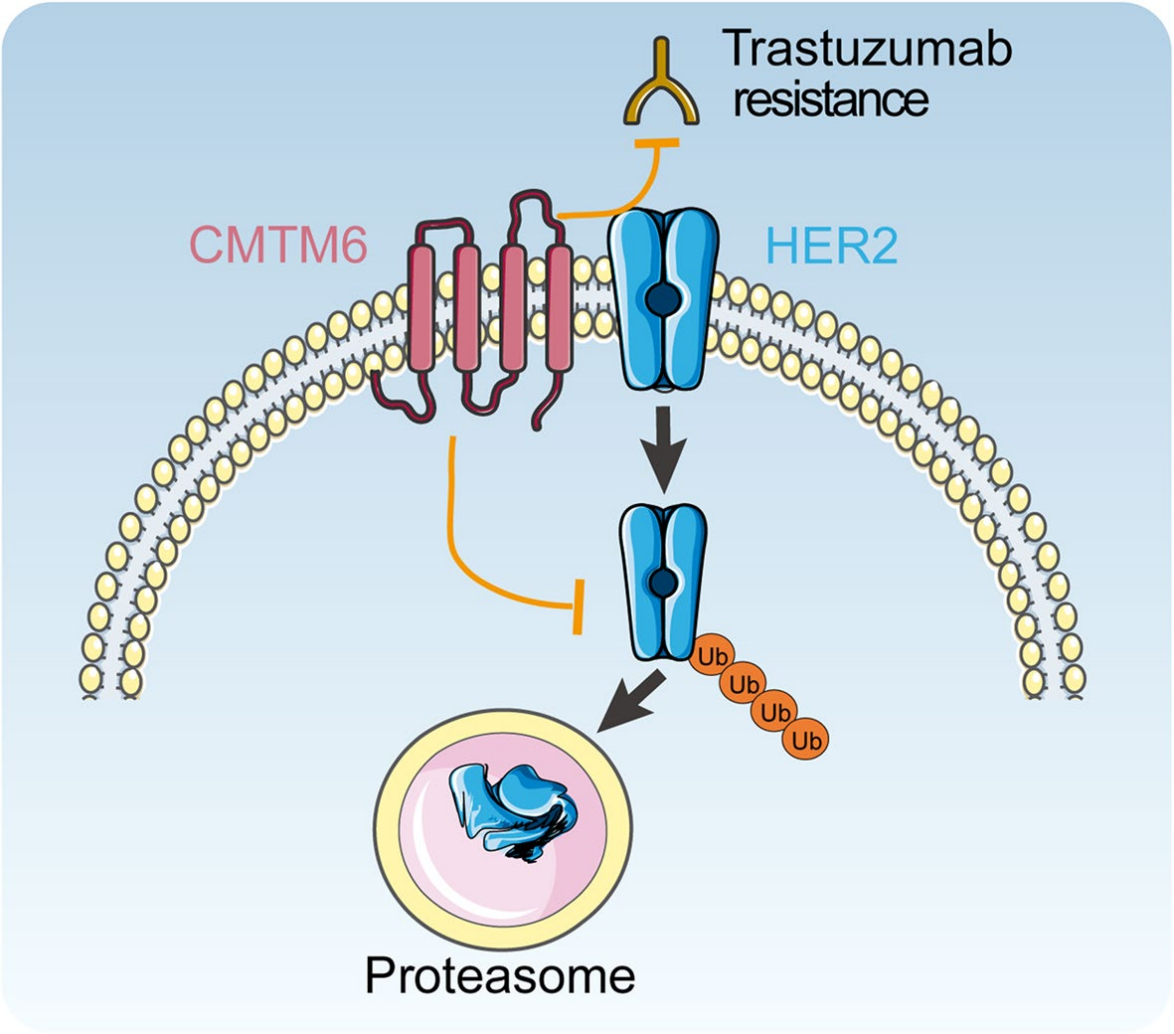
A schematic diagram illustrates the action of CMTM6 in trastuzumab resistance of BC. CMTM6 promotes trastuzumab resistance by inhibiting HER2 ubiquitination and degradation in HER2+ BC

In conclusion, CMTM6 promotes trastuzumab resistance by stabilizing HER2 protein (Fig. 7), and high CMTM6 expression in HER2+ BC is associated with poor prognosis. These novel data suggest that CMTM6 may serve as a prognostic marker to guide clinical decision on using trastuzumab. The addition of a CMTM6 inhibitor to trastuzumab may improve the outcomes of patients with HER2+ BC.

## Supplementary Information

**Additional file 1: Fig. S1.** CMTM6 expression in BC cell lines representing BC clinical subtypes from the Cancer Cell Line Encyclopedia (CCLE) dataset.

**Additional file 2: Fig. S2.** (A, B) GSEA enrichment analysis revealed that the potential signaling pathways related to cell migration, invasion and epithelial to mesenchymal transition (EMT) were positively enriched in high CMTM6 expressing BC. (C, D) Wound healing assay analysis of CMTM6-silenced JIMT-1, CMTM6 overexpressing SKBR3, control JIMT-1 and SKBR3 cells after treatment with 10 μg/ml trastuzumab for 24 h. Data are representative images of each group from three independent experiments.

**Additional file 3: Fig. S3.** Correlation and GSEA enrichment analyses of the relationship between CMTM6 and HER2 expression (A) and the downstream signaling pathways (B) using TCGA-BRCA datasets.

## Data Availability

The data of the relevant dataset in this study can be obtained by contacting the corresponding author.

## Abbreviations

HER2: Human epidermal growth factor receptor 2
CMTM6: CKLF-like MARVEL transmembrane domain-containing 6
BC: Breast cancer
EGF: Epidermal growth factor
MAPK: Mitogen-activated protein kinase
PI3K: Phosphatidylinositol 3′-kinase
PKB/Akt: Protein kinase B
mTOR: Mammalian target of rapamycin
CDK: Cyclin-dependent kinase
PD-L1: Programmed death-ligand 1
GTEx: Genotype-Tissue Expression
TCGA: The Cancer Genome Atlas
BLCA: Bladder urothelial carcinoma
BRCA: Breast invasive carcinoma
COAD: Colon adenocarcinoma
ESCA: Esophageal carcinoma
GBM: Glioblastoma multiforme
KIRP: Kidney renal papillary cell carcinoma
LAML: Acute myeloid leukemia
LGG: Brain lower-grade glioma
LUAD: Lung adenocarcinoma
LUSC: Lung squamous cell carcinoma
OV: Ovarian serous cystadenocarcinoma
PAAD: Pancreatic adenocarcinoma
PRAD: Prostate adenocarcinoma
READ: Rectum adenocarcinoma
STAD: Stomach adenocarcinoma
THCA: Thyroid carcinoma
UCEC: Uterine corpus endometrial carcinoma
UCS: Uterine carcinosarcoma
qRT-PCR: Quantitative real time polymerase chain reaction
CCLE: Cell Cancer Line Encyclopedia
IHC: Immunohistochemistry
OS: Overall survival
RFS: Relapse free survival
CCK-8: Cell Counting Kit-8
EdU: 5-ethynyl-2′-deoxyuridine
TUNEL: Terminal deoxynucleotidyl transferase dUTP nick-end labeling
FITC: Fluorescein isothiocyanate
GSEA: Gene Set Enrichment Analysis
ERK: Extracellular regulated protein kinases
CHX: Cycloheximide
T-DM1: Trastuzumab emtansine
NF-ĸB: Nuclear factor kappa-B
IFN-γ: Interferon gamma
FBS: Fetal bovine serum
DMEM: Dulbeccos Modified Eagle Medium
BSA: Bovine serum albumin
RIPA: Radioimmunoprecipitation
PMSF: Phenylmethane sulfonyl fluoride
SDS-PAGE: Sodium dodecyl sulfate-polyacrylamide gel electrophoresis
PVDF: Polyvinylidene difluoride
TBST: Tris-buffered saline with 0.1% Tween-20
PBS: Phosphate-buffered saline

## References

[1] Sung H, Ferlay J, Siegel RL, Laversanne M, Soerjomataram I, Jemal A, et al. Global cancer statistics 2020: GLOBOCAN estimates of incidence and mortality worldwide for 36 cancers in 185 countries. CA Cancer J Clin. 2021;71:209–49. 33538338 10.3322/caac.21660

[2] Bray F, Ferlay J, Soerjomataram I, Siegel RL, Torre LA, Jemal A. Erratum: global cancer statistics 2018: GLOBOCAN estimates of incidence and mortality worldwide for 36 cancers in 185 countries. CA Cancer J Clin. 2020;70:313–3. 10.3322/caac.21492 30207593

[3] Curtis C, Shah SP, Chin S-F, Turashvili G, Rueda OM, Dunning MJ, et al. The genomic and transcriptomic architecture of 2,000 breast tumours reveals novel subgroups. Nature. 2012;486:346–52. 22522925 10.1038/nature10983 PMC3440846

[4] Slamon DJ, Clark GM, Wong SG, Levin WJ, Ullrich A, McGuire WL. Human breast cancer: correlation of relapse and survival with amplification of the HER-2/neu oncogene. Science (New York, NY). 1987;235:177–82. 10.1126/science.3798106 3798106

[5] Singh K, Tantravahi U, Lomme MM, Pasquariello T, Steinhoff M, Sung CJ. Updated 2013 College of American Pathologists/American Society of Clinical Oncology (CAP/ASCO) guideline recommendations for human epidermal growth factor receptor 2 (HER2) fluorescent in situ hybridization (FISH) testing increase HER2 positive and HER2 equivocal breast cancer cases; retrospective study of HER2 FISH results of 836 invasive breast cancers. Breast Cancer Res Treat. 2016;157:405–11. 27180259 10.1007/s10549-016-3824-x

[6] Li H, Li C, Nan P, Wang T, Wang J, Zhang J, et al. Analysis and validation of PI3K/AKT signaling pathway associated with overall survival time of patients with HER2-positive breast cancer. Med J Chin People Liberation Army. 2018;43:217–23.

[7] Pattanayak B, Lameirinhas A, Torres-Ruiz S, Burgues O, Rovira A, Martinez MT, et al. Role of SALL4 in HER2+ breast Cancer progression: regulating PI3K/AKT pathway. Int J Mol Sci. 2022;23:13292. 36362083 10.3390/ijms232113292 PMC9655635

[8] Patel A, Unni N, Peng Y. The changing paradigm for the treatment of HER2-positive breast Cancer. Cancers. 2020;12:2081. 32731409 10.3390/cancers12082081 PMC7464074

[9] Cameron D, Piccart-Gebhart MJ, Gelber RD. 11 years' follow-up of trastuzumab after adjuvant chemotherapy in HER2-positive early breast cancer: final analysis of the HERceptin adjuvant (HERA) trial (vol 389, pg 1195, 2017). Lancet. 2019;393:1100. 10.1016/S0140-6736(16)32616-2 PMC5465633 28215665

[10] Bergh J, Pritchard KI, Swain S, Cameron D, Albain K, Anderson S, et al. Trastuzumab for early-stage, HER2-positive breast cancer: a meta-analysis of 13 864 women in seven randomised trials. Lancet Oncol. 2021;22:1139–50. 34339645 10.1016/S1470-2045(21)00288-6 PMC8324484

[11] Sakai K, Yokote H, Murakami-Murofushi K, Tamura T, Saijo N, Nishio K. Pertuzumab, a novel HER dimerization inhibitor, inhibits the growth of human lung cancer cells mediated by the HER3 signaling pathway. Cancer Sci. 2007;98:1498–503. 17627612 10.1111/j.1349-7006.2007.00553.x PMC11158426

[12] Scheuer W, Friess T, Burtscher H, Bossenmaier B, Endl J, Hasmann M. Strongly enhanced antitumor activity of Trastuzumab and Pertuzumab combination treatment on HER2-positive human Xenograft tumor models. Cancer Res. 2009;69:9330–6. 19934333 10.1158/0008-5472.CAN-08-4597

[13] Kreutzfeldt J, Rozeboom B, Dey N, De P. The trastuzumab era: current and upcoming targeted HER2+breast cancer therapies. Am J Cancer Res. 2020;10:1045–67. 32368385 PMC7191090

[14] Valabrega G, Montemurro F, Aglietta M. Trastuzumab: mechanism of action, resistance and future perspectives in HER2-overexpressing breast cancer. Ann Oncol. 2007;18:977–84. 17229773 10.1093/annonc/mdl475

[15] Wang H-M, Xu Y-F, Ning S-L, Yang D-X, Li Y, Du Y-J, et al. The catalytic region and PEST domain of PTPN18 distinctly regulate the HER2 phosphorylation and ubiquitination barcodes. Cell Res. 2014;24:1067–90. 25081058 10.1038/cr.2014.99 PMC4152746

[16] Vernieri C, Milano M, Brambilla M, Mennitto A, Maggi C, Cona MS, et al. Resistance mechanisms to anti-HER2 therapies in HER2-positive breast cancer: current knowledge, new research directions and therapeutic perspectives. Crit Rev Oncol Hematol. 2019;139:53–66. 31112882 10.1016/j.critrevonc.2019.05.001

[17] Sanz-Moreno A, Palomeras S, Pedersen K, Morancho B, Pascual T, Galvan P, et al. RANK signaling increases after anti-HER2 therapy contributing to the emergence of resistance in HER2-positive breast cancer. Breast Cancer Res. 2021;23:42. 33785053 10.1186/s13058-021-01390-2 PMC8008631

[18] Gianni L, Pienkowski T, Im Y-H, Roman L, Tseng L-M, Liu M-C, et al. Efficacy and safety of neoadjuvant pertuzumab and trastuzumab in women with locally advanced, inflammatory, or early HER2-positive breast cancer (NeoSphere): a randomised multicentre, open-label, phase 2 trial. Lancet Oncol. 2012;13:25–32. 22153890 10.1016/S1470-2045(11)70336-9

[19] Kong X, Zhang K, Wang X, Yang X, Li Y, Zhai J, et al. Mechanism of trastuzumab resistance caused by HER-2 mutation in breast carcinomas. Cancer Manag Res. 2019;11:5971–82. 31308740 10.2147/CMAR.S194137 PMC6618040

[20] Wu K, Li X, Gu H, Yang Q, Liu Y, Wang L. Research advances in CKLF-like MARVEL Transmembrane domain-containing family in non-small cell lung Cancer. Int J Biol Sci. 2019;15:2576–83. 31754330 10.7150/ijbs.33733 PMC6854381

[21] Guan X, Zhang C, Zhao J, Sun G, Song Q, Jia W. CMTM6 overexpression is associated with molecular and clinical characteristics of malignancy and predicts poor prognosis in gliomas. Ebiomedicine. 2018;35:233–43. 30131308 10.1016/j.ebiom.2018.08.012 PMC6156716

[22] Zhang S, Yan Q, Wei S, Feng X, Xue M, Liu L, et al. CMTM6 and PD-1/PD-L1 overexpression is associated with the clinical characteristics of malignancy in oral squamous cell carcinoma. Oral Surg Oral Med Oral Pathol Oral Radiol. 2021;132:202–9. 34034998 10.1016/j.oooo.2021.02.019

[23] Chen L, Yang Q-C, Li Y-C, Yang L-L, Liu J-F, Li H, et al. Targeting CMTM6 suppresses stem cell-like properties and enhances antitumor immunity in head and neck squamous cell carcinoma. Cancer Immunol Res. 2020;8:179–91. 31771985 10.1158/2326-6066.CIR-19-0394

[24] Zugazagoitia J, Liu Y, Toki M, McGuire J, Ahmed FS, Henick BS, et al. Quantitative assessment of CMTM6 in the tumor microenvironment and association with response to PD-1 pathway blockade in advanced-stage non-small cell lung Cancer. J Thorac Oncol. 2019;14:2084–96. 31605795 10.1016/j.jtho.2019.09.014 PMC6951804

[25] Shang X, Li J, Wang H, Li Z, Lin J, Chen D, et al. CMTM6 is positively correlated with PD-L1 expression and immune cells infiltration in lung squamous carcinoma. Int Immunopharmacol. 2020;88:106864. 32866782 10.1016/j.intimp.2020.106864

[26] Wang H, Gao J, Zhang R, Li M, Peng Z, Wang H. Molecular and immune characteristics for lung adenocarcinoma patients with CMTM6 overexpression. Int Immunopharmacol. 2020;83:106478. 32278132 10.1016/j.intimp.2020.106478

[27] Liu L-L, Zhang S-W, Chao X, Wang C-H, Yang X, Zhang X-K, et al. Coexpression of CMTM6 and PD-L1 as a predictor of poor prognosis in macrotrabecular-massive hepatocellular carcinoma. Cancer Immunol Immunother. 2021;70:417–29. 32770259 10.1007/s00262-020-02691-9 PMC7889680

[28] Burr ML, Sparbier CE, Chan Y-C, Williamson JC, Woods K, Beavis PA, et al. CMTM6 maintains the expression of PD-L1 and regulates anti-tumour immunity. Nature. 2017;549:101–5. 28813417 10.1038/nature23643 PMC5706633

[29] Mezzadra R, Sun C, Jae LT, Gomez-Eerland R, de Vries E, Wu W, et al. Identification of CMTM6 and CMTM4 as PD-L1 protein regulators. Nature. 2017;549:106. 28813410 10.1038/nature23669 PMC6333292

[30] Mishra A, Hourigan D, Lindsay AJ. Inhibition of the endosomal recycling pathway downregulates HER2 activation and overcomes resistance to tyrosine kinase inhibitors in HER2-positive breast cancer. Cancer Lett. 2022;529:153–67. 35007696 10.1016/j.canlet.2022.01.003

[31] Tian Y, Sun X, Cheng G, Ji E, Yang S, Feng J, et al. The association of CMTM6 expression with prognosis and PD-L1 expression in triple-negative breast cancer. Ann Transl Med. 2021;9:131. 33569433 10.21037/atm-20-7616 PMC7867887

[32] Wymant JM, Sayers EJ, Muir D, Jones AT. Strategic Trastuzumab mediated crosslinking driving concomitant HER2 and HER3 endocytosis and degradation in breast Cancer. J Cancer. 2020;11:3288–302. 32231734 10.7150/jca.32470 PMC7097966

[33] Zhao J, Mohan N, Nussinov R, Ma B, Wu WJ. Trastuzumab blocks the receiver function of HER2 leading to the population shifts of HER2-containing Homodimers and heterodimers. Antibodies (Basel). 2021;10:7. 10.3390/antib10010007 PMC7931022 33557368

[34] Magnifico A, Tagliabue E, Ardini E, Casalini P, Colnaghi MI, Menard S. Heregulin beta1 induces the down regulation and the ubiquitin-proteasome degradation pathway of p185HER2 oncoprotein. FEBS Lett. 1998;422:129–31. 9489990 10.1016/s0014-5793(97)01612-8

[35] Hubalek M, Brunner C, Mattha K, Marth C. Resistance to HER2-targeted therapy: mechanisms of trastuzumab resistance and possible strategies to overcome unresponsiveness to treatment. Wien Med Wochenschr. 2010;160:506–12. 20972709 10.1007/s10354-010-0838-6

[36] De Mattos-Arruda L, Cortes J. Use of Pertuzumab for the treatment of HER2-positive metastatic breast Cancer. Adv Ther. 2013;30:645–58. 23881722 10.1007/s12325-013-0043-2

[37] Isakoff SJ, Baselga J. Trastuzumab-DM1: Building a Chemotherapy-Free Road in the Treatment of Human Epidermal Growth Factor Receptor 2 Positive Breast Cancer. J Clin Oncol. 2019;29:351–4. 10.1200/JCO.2010.31.6679 21172881

[38] Xuhong JC, Qi XW, Zhang Y, Jiang J. Mechanism, safety and efficacy of three tyrosine kinase inhibitors lapatinib, neratinib and pyrotinib in HER2-positive breast cancer. Am J Cancer Res. 2019;9:2103–19. 31720077 PMC6834479

