# Correction: CMTM6 overexpression confers trastuzumab resistance in HER2‑positive breast cancer

**DOI:** 10.1186/s12943-023-01918-4

**Published:** 2024-01-05

**Authors:** Fei Xing, Hongli Gao, Guanglei Chen, Lisha Sun, Jiayi Sun, Xinbo Qiao, Jinqi Xue, Caigang Liu

**Affiliations:** Department of Oncology, Innovative Cancer Drug Research and Engineering Center of Liaoning Province, Cancer Stem Cell and Translation Medicine Lab, Shengjing Hospital of China Medical University, Shenyang, 110022 China

Correction: *BMC Microbiol Mol Cancer* **22**, 6 (2023)

10.1186/s12943-023-01716-y

Following publication of the original article [1], the authors reported that they spotted two mistakes in the published version. The two mistakes are, (1) wrong group labels in Fig. 2DD and (2) the orders of image panels in Figure S2C and D were mistaken, the images of wound healing at 0 time point in Supplementary Fig. 2C were highly similar between the shRNA#1 and shRNA#2 groups. In addition, the image of the CMTM6 group at 24 h in Supplementary Fig. 2D was easily misunderstood due to the off-center position of the scratch shown. To ensure its accuracy, they wish to correct these errors by replacing correct images in Figure S2C and D, and re-arranging the orders of images in Figure S2C and D as well as correcting the group labels in Fig. 2D. The corrections do not alter the findings and conclusions of the study.

The original article [1] has been corrected.
