# Supplementary figures and images for: CMTM6 overexpression confers trastuzumab resistance in HER2-positive breast cancer
Source: Mol Cancer. 2023 Jan 10;22:6. doi: 10.1186/s12943-023-01716-y (PMC9830830; doi:10.1186/s12943-023-01716-y)

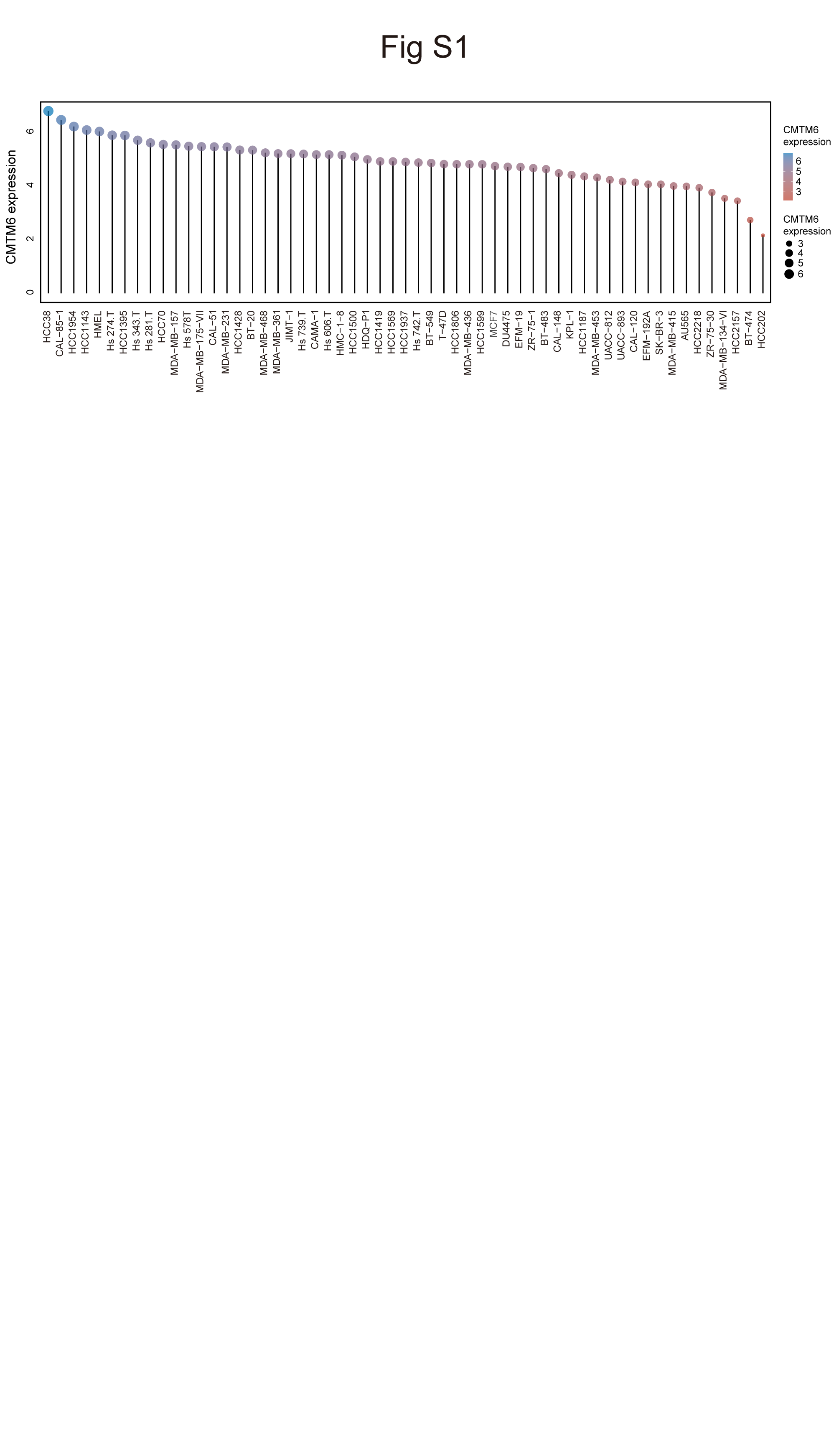

Supplement: Supplementary file 1 — Additional file 1: Fig. S1. CMTM6 expression in BC cell lines representing BC clinical subtypes from the Cancer Cell Line Encyclopedia (CCLE) dataset. [file 12943_2023_1716_MOESM1_ESM.tif]

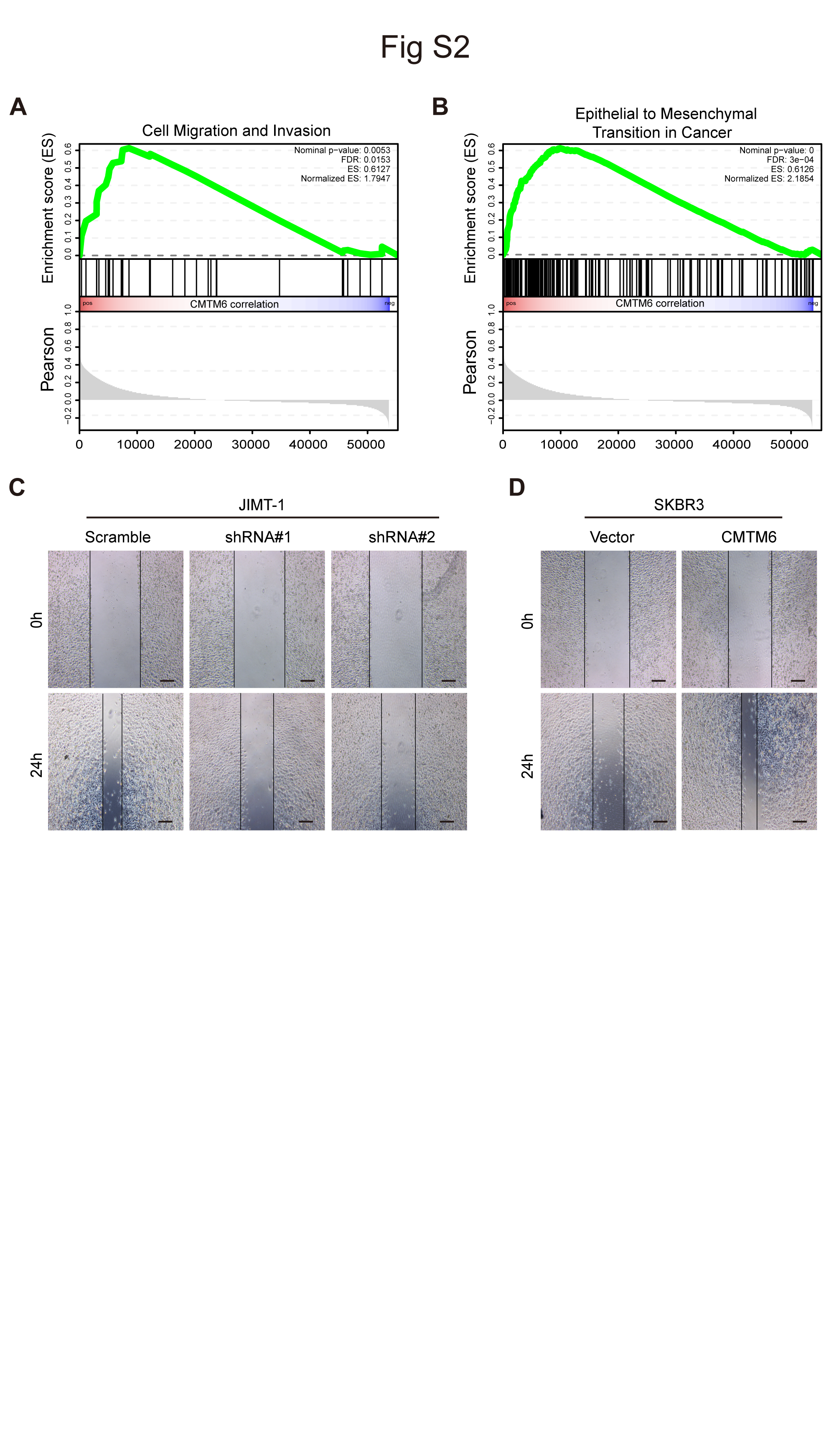

Supplement: Supplementary file 2 — Additional file 2: Fig. S2. (A, B) GSEA enrichment analysis revealed that the potential signaling pathways related to cell migration, invasion and epithelial to mesenchymal transition (EMT) were positively enriched in high CMTM6 expressing BC. (C, D) Wound healing assay analysis of CMTM6-silenced JIMT-1, CMTM6 overexpressing SKBR3, control JIMT-1 and SKBR3 cells after treatment with 10 μg/ml trastuzumab for 24 h. Data are representative images of each group from three independent experiments. [file 12943_2023_1716_MOESM2_ESM.tif]

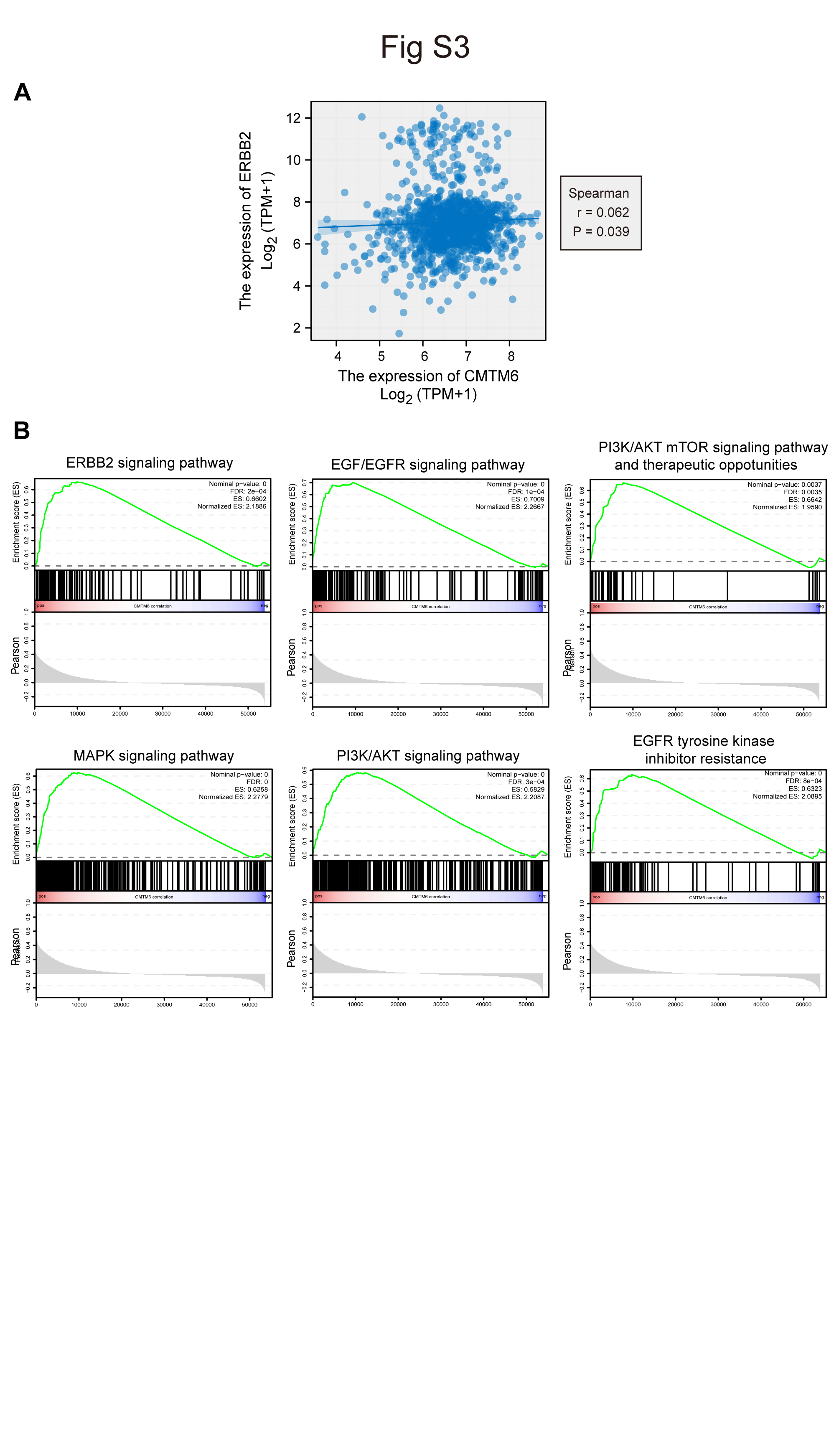

Supplement: Supplementary file 3 — Additional file 3: Fig. S3. Correlation and GSEA enrichment analyses of the relationship between CMTM6 and HER2 expression (A) and the downstream signaling pathways (B) using TCGA-BRCA datasets. [file 12943_2023_1716_MOESM3_ESM.tif]
